# Enantiocomplementary Michael Additions of Acetaldehyde to Aliphatic Nitroalkenes Catalyzed by Proline‐Based Carboligases

**DOI:** 10.1002/cbic.202100644

**Published:** 2022-02-02

**Authors:** Andreas Kunzendorf, Mohammad Saifuddin, Gerrit J. Poelarends

**Affiliations:** ^1^ Department of Chemical and Pharmaceutical Biology Groningen Research Institute of Pharmacy University of Groningen Antonius Deusinglaan 1 9713 AV Groningen The Netherlands; ^2^ Molecular Enzymology Group University of Groningen Nijenborgh 4 9747 AG Groningen The Netherlands

**Keywords:** biocatalysis, enamine catalysis, enzyme catalysis, Michael addition, nitroaldehydes

## Abstract

The blockbuster drug Pregabalin is widely prescribed for the treatment of painful diabetic neuropathy. Given the continuous epidemic growth of diabetes, the development of sustainable synthesis routes for Pregabalin and structurally related pharmaceutically active γ‐aminobutyric acid (GABA) derivatives is of high interest. Enantioenriched γ‐nitroaldehydes are versatile synthons for the production of GABA derivatives, which can be prepared through a Michael‐type addition of acetaldehyde to α,β‐unsaturated nitroalkenes. Here we report that tailored variants of the promiscuous enzyme 4‐oxalocrotonate tautomerase (4‐OT) can accept diverse aliphatic α,β‐unsaturated nitroalkenes as substrates for acetaldehyde addition. Highly enantioenriched aliphatic (*R*)‐ and (*S*)‐γ‐nitroaldehydes were obtained in good yields using two enantiocomplementary 4‐OT variants. Our results underscore the synthetic potential of 4‐OT for the preparation of structurally diverse synthons for bioactive analogues of Pregabalin.

## Graphical abstract

**Enantiocomplementary Michael additions**: The application of two tailor‐made enantiocomplementary carboligases allow for the stereodivergent synthesis of aliphatic γ‐nitroaldehydes, which are useful precursors for pharmaceutically active analogues of the blockbuster drug, Pregabalin. Good isolated product yields and excellent enantiopurity (up to >99 : 1 e.r.) were achieved.

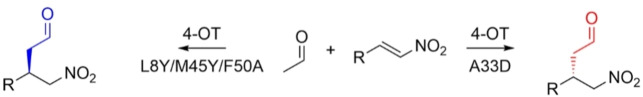

## Introduction

**Scheme 1:**
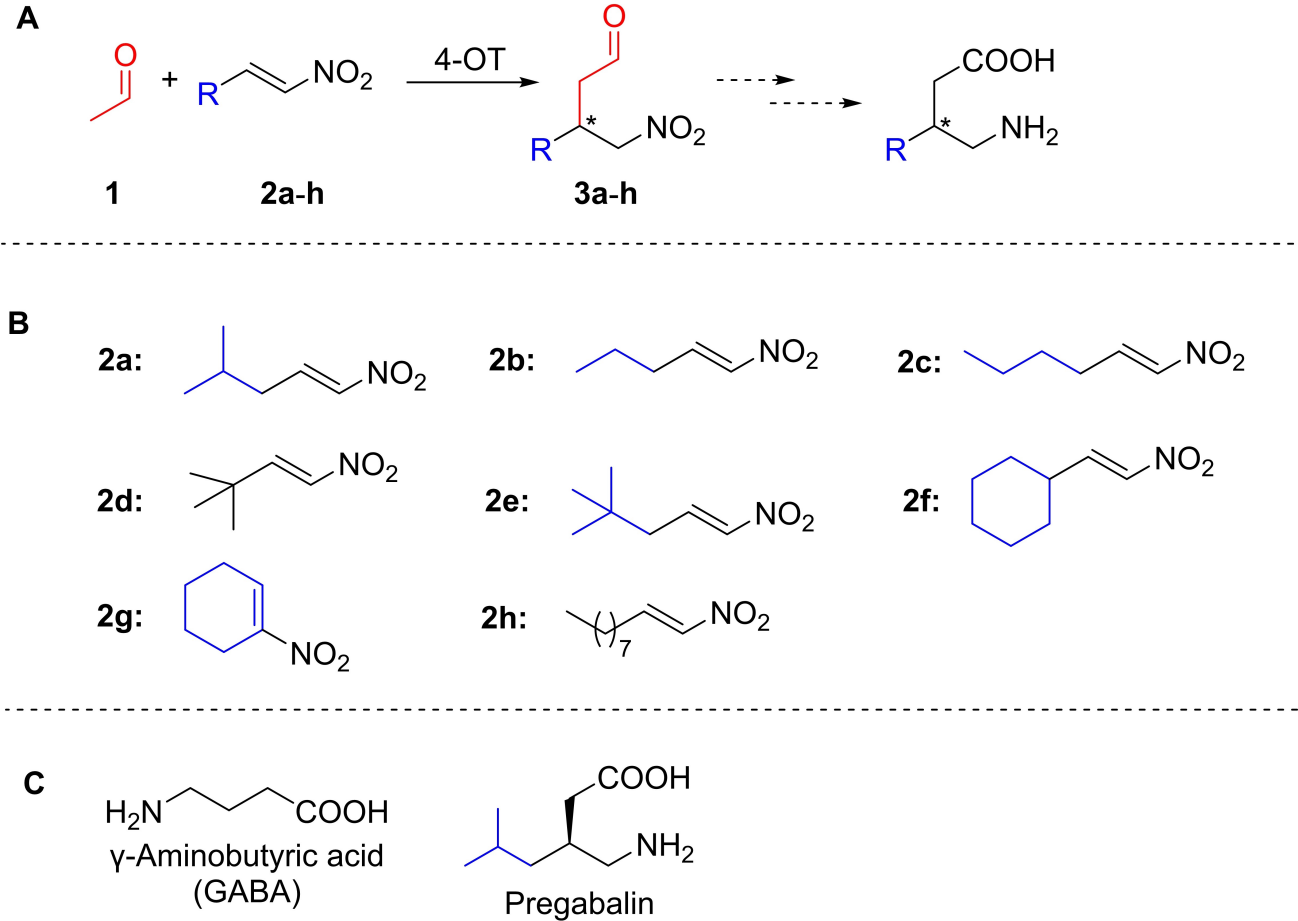
A) Michael‐type addition of acetaldehyde **1** to α,β‐unsaturated nitroalkenes **2** catalyzed by 4‐OT to yield enantioenriched γ‐nitroaldehydes **3** as precursors for valuable γ‐aminobutyric acids (GABA) derivatives. B) Chemical structures of the nitroalkenes **2 a‐2 h**. C) Chemical structures of GABA and its derivative Pregabalin.

Chiral γ‐nitroaldehydes are versatile building blocks for γ‐aminobutyric acid (GABA, Scheme 1) derivatives that have broad application as pharmaceuticals, such as Pregabalin (anticonvulsant, Scheme 1), Rolipram (antidepressant) or Baclofen (muscle relaxant).[^1^, ^2^, ^3^] The blockbuster drug Pregabalin is widely prescribed for the treatment of painful diabetic neuropathy,^[4]^ and with the continuous epidemic growth of diabetes, the development of efficient and sustainable synthesis routes for Pregabalin and structurally related pharmaceutically active GABA derivatives is of high academic and industrial interest.

**Figure 1:**
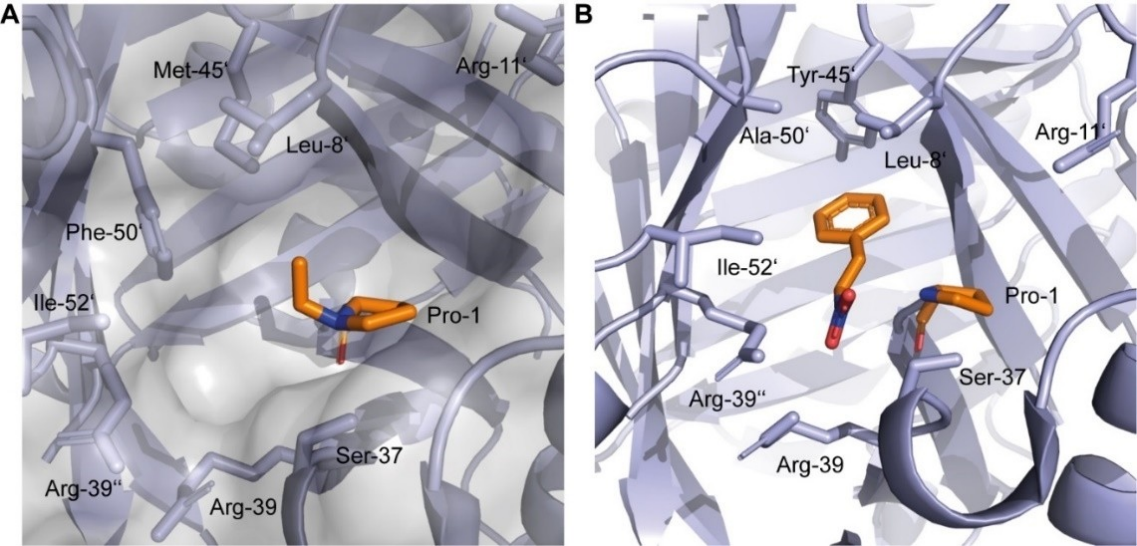
Crystal structures of different enzyme‐substrate complexes. A) Active site of 4‐OT wild‐type (PDB: 4X1C) with acetaldehyde covalently bound as an enamine intermediate to the N‐terminal proline (Pro‐1). B) Crystal structure of 4‐OT M45Y/F50A (PDB: 5CLO) with an α,β‐unsaturated nitroalkene (nitrostyrene) bound in the active site.

The addition of acetaldehyde to α,β‐unsaturated nitroalkenes offers convenient access to γ‐nitroaldehydes from which the respective GABA derivatives can be obtained in two simple chemical steps.^[5]^ Consequently, extensive research has been focused on the asymmetric synthesis of γ‐nitroaldehydes mainly through using proline‐derived organocatalysts or small peptides.[^6^, ^7^, ^8^, ^9^] We reported that the enzyme 4‐oxalocrotonate tautomerase (4‐OT) is able to promiscuously catalyze the addition of acetaldehyde **1** to several α,β‐unsaturated nitroalkenes **2**, yielding the corresponding γ‐nitroaldehydes **3** with moderate to good enantiopurity (Scheme 1).[^10^, ^11^] 4‐OT is a homohexameric enzyme that belongs to the tautomerase superfamily and naturally utilizes its N‐terminal proline (Pro‐1) as a base to catalyze the enol‐keto tautomerisation of 2‐hydroxymuconate.[^12^, ^13^] Comprehensive mechanistic and crystallographic studies suggest that the 4‐OT catalyzed Michael‐type additions proceed through Pro‐1 dependent activation of acetaldehyde to give a reactive enzyme‐bound enamine intermediate, which reacts with an α,β‐unsaturated nitroalkene to yield, upon hydrolysis, a γ‐nitroaldehyde (Figure 1).[^10^, ^14^]

Guided by mutability landscapes that revealed hotspot positions for improved activity and stereoselectivity, the synthetic usefulness of 4‐OT for the stereodivergent synthesis of γ‐nitroaldehydes has been optimized by protein engineering.^[15]^ Using the two tailor‐made enantiocomplementary 4‐OT variants M45Y/F50A and A33D, enzymatic access to both enantiomers of several γ‐nitroaldehydes was achieved. Subsequently, the enantioselectivity of variant 4‐OT M45Y/F50A was further optimized by structure‐inspired (Figure 1B) protein engineering resulting in variant 4‐OT L8Y/M45Y/F50A, which promotes the asymmetric synthesis of the pharmaceutically relevant enantiomers of several γ‐nitroaldehydes with excellent stereoselectivity.^[16]^

In previous work, we have demonstrated that wild‐type 4‐OT and engineered 4‐OT variants accept various aromatic α,β‐unsaturated nitroalkenes in Michael‐type addition reactions with acetaldehyde.[^10^, ^11^, ^15^, ^16^, ^17^] In contrast, only one aliphatic α,β‐unsaturated nitroalkene as substrate for this 4‐OT catalyzed reaction has been reported, yielding the γ‐nitroaldehyde precursor for Pregabalin. Structural analogues of Pregabalin show a diverse effect on biological activity,^[1]^ which emphasizes the importance of developing efficient synthetic strategies for related aliphatic GABA derivatives and their precursors. In this study, we therefore focused on further expanding the substrate scope of the enantiocomplementary 4‐OT variants L8Y/M45Y/F50A and A33D towards aliphatic α,β‐unsaturated nitroalkenes as Michael acceptors, enabling the asymmetric synthesis of various linear, branched and cyclic aliphatic γ‐nitroaldehydes.

## Results and Discussion

**Scheme 2:**
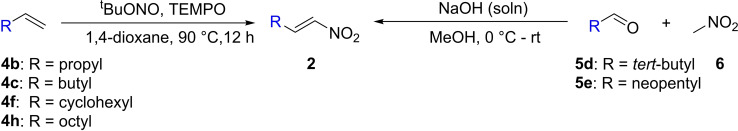
Chemical synthesis of aliphatic α,β‐unsaturated nitroalkenes **2**. The applied procedures yielded the (*E*)‐isomers as the major products, which were purified by silica gel column chromatography.

We started our investigation by preparing a set of aliphatic α,β‐unsaturated nitroalkenes (**2 b**–**f**, **2 h**) with different alkyl substitutions connected to the carbon‐carbon double bond, either by a nitration of olefins **4** with ^t^BuONO and TEMPO,^[18]^ or by a Henry reaction, followed by dehydration, between aldehydes **5** and nitromethane **6** (Scheme 2, see Supporting Information for details).

The chemically prepared nitroalkenes **2 b**–**f**, **2 h**, as well as commercially available **2 g**, were then tested as potential Michael acceptors in acetaldehyde (**1**) additions catalyzed by 4‐OT variant A33D or L8Y/M45Y/F50A. To prevent undesired non‐enzymatic water addition to the α,β‐unsaturated nitroalkenes, the reactions for the addition of **1** (150 mM) to **2 b**–**h** (3 mM) were performed in sodium phosphate buffer at pH 6.5 and with suitable biocatalyst loading (100 μM) to shorten reaction times.^[11]^ Up to 15 % ethanol was used as a cosolvent to solubilize the relatively apolar aliphatic α,β‐unsaturated nitroalkenes **2 b**–**h**. Previous experiments indicated that ethanol, in contrast to other cosolvents such as DMSO, has no negative effect on the stereoselectivity of 4‐OT.^[16]^ The progress of the semi‐preparative scale reactions was followed by UV spectroscopy until the decrease in absorbance at λ=250 nm indicated completion of the reactions. The reaction times for the conversion of **2 b**–**f** ranged from <0.5 h to 2.5 h.

Gratifyingly, both 4‐OT variants readily accepted α,β‐unsaturated nitroalkenes **2 b**–**f** as non‐native substrates to give the corresponding γ‐nitroaldehyde products **3 b**–**f** in good to excellent yields (46 %–92 %, Figure 2, see Supporting Information Figures S1‐32). The sterically more demanding nitroalkenes **2 g**, **h** were not accepted as substrates (<1 % conversion after 2.5 h reaction time). Thus, both 4‐OT variants accept linear, branched and cyclic aliphatic α,β‐unsaturated nitroalkenes as acceptors for the Michael‐type addition of **1**.

**Figure 2:**
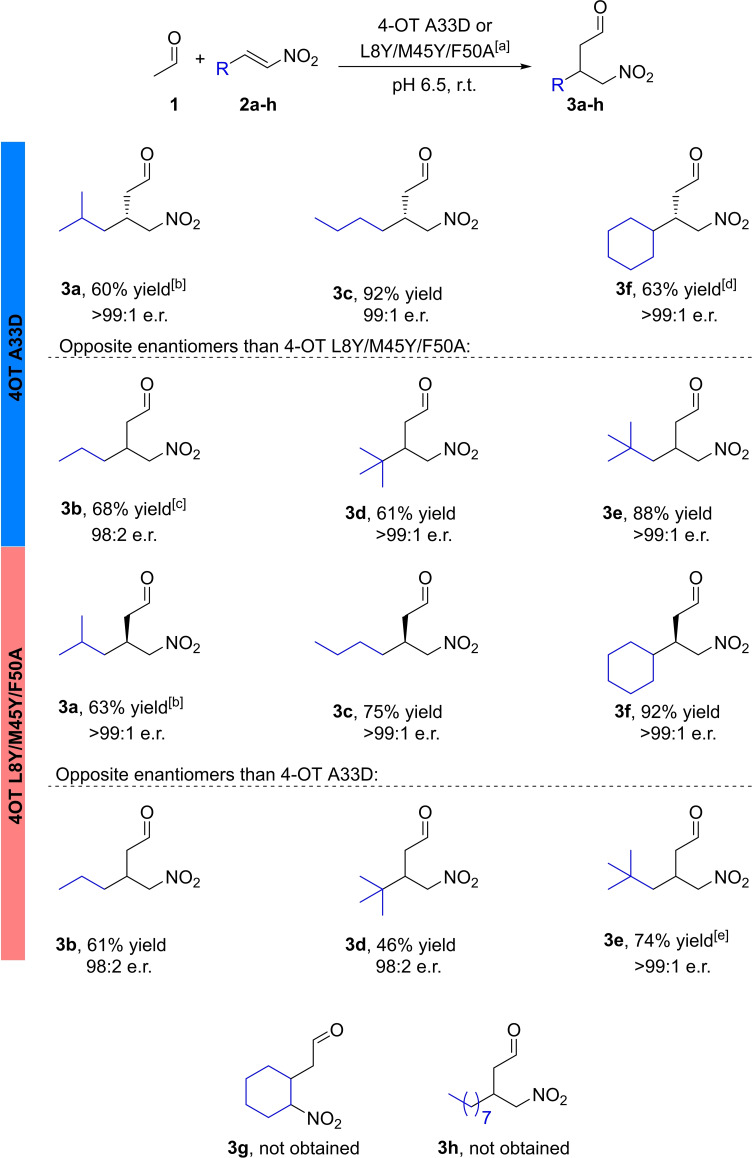
Enantiocomplementary synthesis of enantioenriched γ‐nitroaldehydes catalyzed by 4‐OT A33D or 4‐OT L8Y/M45Y/F50A. [a] Reaction conditions: 150 mM acetaldehyde **1**, 3 mM nitroalkene **2 a**–**f**, 100 μM 4‐OT variant, 15 % ethanol in 20 mM NaH_2_PO_4_, pH 6.5. Yields are isolated yields; the e.r. was determined by chiral‐phase GC. [b] Reported previously.[^15^, ^16^] [c] 120 μM 4‐OT A33D. [d] Further purified using column chromatography. [e] 10 % ethanol as a cosolvent.

The enantiomeric ratio and absolute configuration of the products were determined by chiral‐phase GC analysis using chemically prepared racemic **3 b**–**f** as reference compounds. Analysis of the products showed that variants 4‐OT A33D and 4‐OT L8Y/M45Y/F50A afford the respective enantiomers of **3 b**–**f** with excellent enantiopurity (e.r. up to >99 : 1, see Supporting Information Figures S1–32). For products **3 c**, **f** the absolute configuration was determined by comparison with previously reported literature data.[^6^, ^7^] Variant 4‐OT A33D exclusively yielded the (*R*)‐enantiomer of compound **3 c** and the (*S*)‐enantiomer of product **3 f** (same geometry but different priority of the substituents at the chiral center). Indeed, variant 4‐OT L8Y/M45Y/F50A afforded exclusively the opposite enantiomers, that is (*S*)‐**3 c** and (*R*)‐**3 f**. For products **3 b**, **d**, **e** the absolute configuration was not determined, but again opposite enantiomers were produced by 4‐OT A33D and 4‐OT L8Y/M45Y/F50A. Thus, convenient enantiocomplementary synthesis of the (*R*)‐ and (*S*)‐enantiomers of **3 b**–**f** can be achieved by using these tailor‐made 4‐OT variants. Pleasingly, the stereopreference and high enantioselectivity of both 4‐OT variants, as previously observed with aromatic compounds, is also witnessed with the aliphatic substrates. Taken together, these results demonstrate an attractive biocatalytic approach for the asymmetric synthesis of highly enantioenriched synthons for pharmaceutically active aliphatic GABA derivatives.

## Conclusion

In conclusion, we have explored the substrate scope of two previously engineered 4‐OT variants[^15^, ^16^] using a focused set of linear, branched, and cyclic aliphatic α,β‐unsaturated nitroalkenes. Biocatalytic reactions using acetaldehyde **1** as a substrate are scarce and often face challenges due to the high inherent reactivity of **1**.[^19^, ^20^] Pleasingly, the here presented 4‐OT catalyzed Michael reactions with **1** as a nucleophile readily proceed under mild conditions in an environmentally friendly aqueous buffer system with ethanol as a green and sustainable cosolvent.^[21]^ In contrast to aromatic nitroalkenes,[^22^, ^23^] aliphatic nitroalkenes are prone to rapid non‐enzymatic hydration. To outcompete this undesired hydration of the nitroalkenes **2 a**–**h**, we kept reaction times short by using a rather high catalyst loading as well as an excess of acetaldehyde **1**. In comparison with previously reported procedures for the preparation of γ‐nitroaldehydes using small‐molecule aminocatalysts, the here presented enzymatic reactions proceed in environmentally benign aqueous solvent and use up to 6‐times lower catalyst loading.[^6^, ^7^, ^8^, ^9^]

Both assessed 4‐OT variants successfully catalyze the Michael‐type addition of acetaldehyde **1** to several aliphatic α,β‐unsaturated nitroalkenes **2 b**–**f** yielding both enantiomers of the corresponding γ‐nitroaldehydes **3 b‐**‐**f** with good yields and excellent enantiopurity (e.r.: 98 : 2 to >99 : 1). Notably, the products **3 b**–**f** are valuable precursors for the respective GABA derivatives, which show bioactivity as anticonvulsants in pharmacokinetic *in vitro* assays and animal studies.[^24^, ^25^]

Using previously developed chemoenzymatic cascade reactions, the prepared γ‐nitroaldehydes can be readily converted in two steps into the respective GABA analogous.^[16]^ Together with our continued protein engineering efforts to improve 4‐OT's stability and activity,[^26^, ^27^] this work further highlights the potential of proline‐based carboligases as powerful catalysts for abiological carbon‐carbon bond‐forming reactions.

## Experimental Section

A detailed description of the experimental methods and product characterization can be found in the Supporting Information.

## Conflict of interest

The authors declare no conflict of interest.

1

## Supporting information

As a service to our authors and readers, this journal provides supporting information supplied by the authors. Such materials are peer reviewed and may be re‐organized for online delivery, but are not copy‐edited or typeset. Technical support issues arising from supporting information (other than missing files) should be addressed to the authors.

Supporting Information

## Data Availability

The data that support the findings of this study are available in the supplementary material of this article.
