# Supplementary material for: Enantiocomplementary Michael Additions of Acetaldehyde to Aliphatic Nitroalkenes Catalyzed by Proline‐Based Carboligases
Source: Chembiochem. 2022 Feb 2;23(6):e202100644. doi: 10.1002/cbic.202100644 (PMC9306545; doi:10.1002/cbic.202100644)
Supplement: Supplementary file 1 — Supporting Information [file CBIC-23-0-s001.pdf]

ChemBioChem

Supporting Information

Enantiocomplementary Michael Additions of Acetaldehyde to Aliphatic Nitroalkenes Catalyzed by Proline-Based Carboligases

Andreas Kunzendorf[+], Mohammad Saifuddin[+], and Gerrit J. Poelarends*

Contents

General methods 2

Synthesis of nitroalkenes 3

General procedure for the synthesis of compounds **2b**,**c**,**f**,**h** 3

General procedure for the synthesis of compounds **2d**,**e** 4

Synthesis of racemic reference compounds **3b-f** 5

Expression and purification of 4-OT 11

Enzymatic semi-preparative scale reactions for **3b-f** 12

Supplementary references 33

General methods

Chemicals, including nitroalkene **2g**, were obtained from Sigma-Aldrich Chemical Co. (St. Louis, MO, USA) or TCI Europe N.V. (Zwijndrecht, Belgium) unless otherwise stated. Organic solvents were purchased from Biosolve (Valkenswaard, The Netherlands) and Sigma-Aldrich Chemical Co. Spectrophotometric measurements were performed on a V-650 or V-660 spectrophotometer from Jasco (IJsselstein, The Netherlands). Chiral-phase GC analysis was performed with a Shimadzu GC-2010 gas chromatograph on an Astec CHIRALDEX G-TA column. High resolution mass spectrometry (HRMS) data was obtained from the Mass Spectrometry core facility of the University of Groningen. NMR spectra were recorded on a Bruker DRX-500 (500 MHz) spectrometer at the Drug Design laboratory of the University of Groningen. Chemical shifts (δ) are reported in parts per million (ppm) and are referenced to $CHCl_3$ (δ = 7.26 ppm).

Synthesis of nitroalkenes

General procedure for the synthesis of compounds **2b**,**c**,**f**,**h**

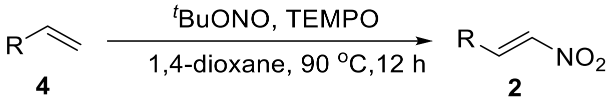

To an oven-dried round-bottom flask charged with a magnetic stir-bar was added TEMPO (0.4 equiv.), olefin (**4**) (1.0 equiv.), ^{t}BuONO (2.0 equiv.) and 1,4-dioxane (20 mL).[1] After the addition of all the starting material, the mixture was stirred at 90 °C for 12 h. After the completion of starting material (monitored by TLC), the reaction mixture was cooled to room temperature followed by work-up using EtOAc/H_2O (3 x 20 mL). The organic extracts were combined, dried over Na_2SO_4, and evaporated using a rotary evaporator. The crude products were further purified by silica gel column chromatography (using petroleum ether/ethyl acetate 95:5) as an eluent to give the corresponding nitroalkenes **2**.

(*E*)-1-nitropent-1-ene (**2b**): Pale yellow liquid; yield = 35% (575 mg, starting from 1.0 g of **4b**).

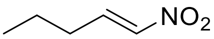

The ^{1}H NMR data of compound **2b** match with earlier reported NMR data.[2] ^{1}H NMR (500 MHz, $CDCl_3$) δ 7.31 – 7.24 (m, 1H), 6.98 (dd, *J* = 13.4, 1.4 Hz, 1H), 2.25 (qd, *J* = 7.4, 1.5 Hz, 2H), 1.56 (q, *J* = 7.4 Hz, 2H), 0.98 (t, *J* = 7.4 Hz, 3H).

(*E*)-1-nitrohex-1-ene (**2c**): Pale yellow liquid; yield = 41% (630 mg, starting from 1.0 g of **4c**).

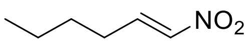

The ^{1}H NMR data of compound **2c** match with earlier reported NMR data.[3] ^{1}H NMR (500 MHz, $CDCl_3$) δ 7.32 – 7.25 (m, 1H), 6.99 (d, *J* = 13.4 Hz, 1H), 2.33 – 2.22 (m, 2H), 1.55 – 1.47 (m, 2H), 1.39 (dq, *J* = 14.4, 7.2 Hz, 2H), 0.94 (t, *J* = 7.3 Hz, 3H).

(*E*)-(2-nitrovinyl)cyclohexane (**2f**): Pale yellow liquid; yield = 50% (705 mg, starting from 1.0 g of **4f**).

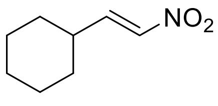

The ^{1}H NMR data of compound **2f** match with earlier reported NMR data.[4] ^{1}H NMR (500 MHz, $CDCl_3$) δ 7.22 (dd, *J* = 13.5, 7.2 Hz, 1H), 6.93 (d, *J* = 13.5 Hz, 1H), 2.25 (dtd, *J* = 11.0, 7.6, 3.5 Hz, 1H), 1.85 – 1.76 (m, 4H), 1.74 – 1.68 (m, 1H), 1.38 – 1.28 (m, 2H), 1.26 – 1.15 (m, 3H).

(*E*)-1-nitrodec-1-ene (**2h**): Pale yellow liquid; yield = 38% (501 mg, starting from 1.0 g of **4h**).

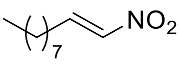

The ^{1}H NMR data of compound **2h** match with earlier reported NMR data.[5] ^{1}H NMR (500 MHz, $CDCl_3$) δ 7.31 – 7.24 (m, 1H), 7.00 – 6.95 (m, 1H), 2.26 (q, *J* = 7.3 Hz, 2H), 1.51 (p, *J* = 7.3 Hz, 2H), 1.35 – 1.22 (m, 10H), 0.88 (t, *J* = 6.8 Hz, 3H).

General procedure for the synthesis of compounds **2d**,**e**

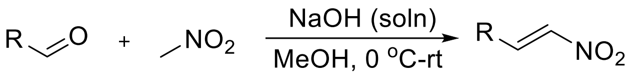

To a mixture of aldehyde (**5**) (1.0 equiv.) and nitromethane (**6**) (1.0 equiv.) in methanol (10 mL) at 0 °C, a solution of NaOH in H_2O (1.2 equiv.) was added dropwise. Further methanol (2 mL) was added and the resulting yellow slurry stirred at 0 °C for 1 h. Water (30 mL) was added and the clear yellow solution was poured into hydrochloric acid (20 mL conc. hydrochloric acid in 30 mL H_2O) and stirred for 15 min. The aqueous mixture was extracted with DCM (10 mL x 3), the combined organic layers dried over sodium sulfate, and the solvent removed using a rotary evaporator. The residue was purified by column chromatography (using petroleum ether/ethyl acetate 95:5) to give the nitro-olefins **2**.

(*E*)-3,3-dimethyl-1-nitrobut-1-ene (**2d**): Pale yellow liquid; yield = 31% (465 mg, starting from 1.0 g of **5d**). The ^{1}H NMR data of compound **2d** match with earlier reported NMR data.[4] ^{1}H NMR (500 MHz, $CDCl_3$) δ 7.26 (d, J = 13.6 Hz, 1H), 6.90 (d, J = 13.6 Hz, 1H), 1.16 (s, 9H).

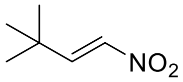

(*E*)-4,4-dimethyl-1-nitropent-1-ene (**2e**): Pale yellow liquid; yield = 39% (560 mg, starting from 1.0 g of **5e**). ^{1}H NMR (500 MHz, $CDCl_3$) δ 7.34 – 7.24 (m, 1H), 6.97 (dt, J = 13.3, 1.2 Hz, 1H), 2.14 (dd, J = 8.4, 1.3 Hz, 2H), 0.98 (s, 9H). ^{13}C NMR (126 MHz, $CDCl_3$) δ 140.61, 140.40, 42.44, 31.69, 29.34. HRMS (ESI+): calcd. for $C_7H_{13}NO_2$ [M+H]+: 144.1019, found: 144.1019.

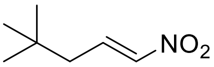

Synthesis of racemic reference compounds **3b-f**

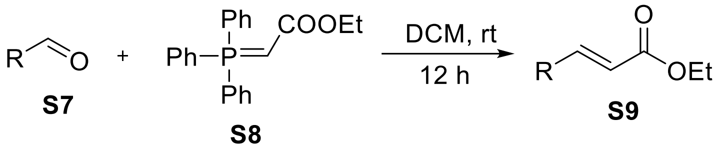

Aldehyde (**S7**) (1.0 equiv.) was dissolved in 5 mL anhydrous DCM and treated with carbethoxy-methylene triphenylphosphorane (**S8**) (1.1 equiv.) at room temperature. After the complete addition of all the starting material, the mixture was stirred at rt for 12 h. After the completion of starting material (monitored by TLC, $KMnO_4$ staining), the reaction mixture was quenched with saturated aqueous ammonium chloride, and extracted 3 times with DCM. The organic extracts were combined, dried over Na_2SO_4, and evaporated using a rotary evaporator. The crude products were further purified by silica gel column chromatography (using petroleum ether/ethyl acetate 95:5) as an eluent to give the corresponding α,β–unsaturated ester **S9**.

Ethyl (*E*)-hex-2-enoate (**S9b**): clear oil; yield = 76% (300 mg, starting from 200 mg of **S7b**).

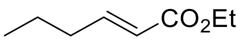

The ^{1}H NMR data of compound **S9b** match with earlier reported NMR data.[6] ^{1}H NMR (500 MHz, $CDCl_3$) δ 6.96 (dt, *J* = 15.6, 7.0 Hz, 1H), 5.81 (dt, *J* = 15.6, 1.5 Hz, 1H), 4.18 (q, *J* = 7.1 Hz, 2H), 2.18 (qd, *J* = 7.2, 1.5 Hz, 2H), 1.49 (h, *J* = 7.4 Hz, 2H), 1.29 (t, *J* = 7.1 Hz, 3H), 0.94 (t, *J* = 7.4 Hz, 3H).

Ethyl (*E*)-hept-2-enoate (**S9c**): clear oil; yield = 80% (290 mg, starting from 200 mg of **S7c**).

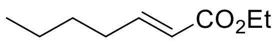

The ^{1}H NMR data of compound **S9c** match with earlier reported NMR data.[7] ^{1}H NMR (500 MHz, $CDCl_3$) δ 7.01 – 6.92 (m, 1H), 5.81 (dt, *J* = 15.6, 1.5 Hz, 1H), 4.18 (q, *J* = 7.1 Hz, 2H), 2.20 (qd, *J* = 7.3, 1.5 Hz, 2H), 1.44 (ddd, *J* = 12.2, 8.5, 6.1 Hz, 2H), 1.35 (dt, *J* = 14.8, 7.1 Hz, 2H), 1.28 (t, *J* = 7.1 Hz, 3H), 0.91 (t, *J* = 7.3 Hz, 3H).

Ethyl (*E*)-5,5-dimethylhex-2-enoate (**S9e**): clear oil; yield = 41% (139 mg, starting from 200 mg of **S7e**).

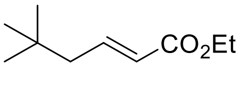

The ^{1}H NMR data of compound **S9e** match with earlier reported NMR data.[8] ^{1}H NMR (500 MHz, $CDCl_3$) δ 6.98 (dt, *J* = 15.7, 7.9 Hz, 1H), 5.80 (dt, *J* = 15.5, 1.3 Hz, 1H), 4.19 (q, *J* = 7.1 Hz, 2H), 2.08 (dd, *J* = 7.9, 1.3 Hz, 2H), 1.29 (t, *J* = 7.1 Hz, 3H), 0.93 (s, 9H).

Ethyl (*E*)-3-cyclohexylacrylate (**S9f**): clear oil; yield = 50% (227 mg, starting from 200 mg of **S7f**).

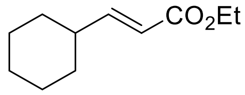

The ^{1}H NMR data of compound **S9f** match with earlier reported NMR data.[9] ^{1}H NMR (500 MHz, $CDCl_3$) δ 6.88 (dd, *J* = 15.8, 6.8 Hz, 1H), 5.73 (dd, *J* = 15.8, 1.3 Hz, 1H), 4.15 (q, *J* = 7.1 Hz, 2H), 2.14 – 2.04 (m, 1H), 1.72 (dq, *J*

= 9.1, 3.3 Hz, 4H), 1.68 – 1.60 (m, 1H), 1.31 – 1.21 (m, 5H), 1.13 (dtd, *J* = 16.5, 12.2, 9.5 Hz, 3H).

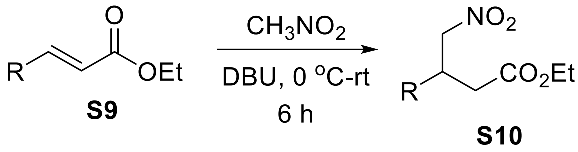

To a stirred solution of α,β–unsaturated ester (**S9**) (1.0 equiv.) in nitromethane **6** (5.0 equiv.) cooled at 0 °C, was added dropwise, under nitrogen, (1.0 equiv.) of 1.8-diazabicyclo[5.4.0]undec-7-ene (DBU). The reaction mixture was incubated at room temperature for 6 h. After the completion of starting material (monitored by TLC, $KMnO_4$ staining), the reaction was quenched with a 1 M aqueous hydrogen chloride solution. The solution was extracted with diethyl ether (5 ml, 3 times). The organic extracts were combined, dried over Na_2SO_4, and evaporated using a rotary evaporator. The crude products were further purified by silica gel column chromatography (using petroleum ether/ethyl acetate from 95:5 to 90:10) as an eluent to give the corresponding Michael adducts **S10**.

Ethyl 3-(nitromethyl)hexanoate (**S10b**): Light pale yellow oil; yield = 82% (117 mg, starting

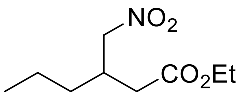

from 100 mg of **S9b**). The ^{1}H NMR data of compound **S10b** match with earlier reported NMR data.[10] ^{1}H NMR (500 MHz, $CDCl_3$) δ 4.56 – 4.40 (m, 2H), 4.15 (q, *J* = 7.1 Hz, 2H), 2.64 (p, *J* = 6.4 Hz, 1H), 2.43 (d, *J* = 6.4 Hz, 2H), 1.39 (dq, *J* = 9.4, 6.5, 4.6 Hz, 4H), 1.27 (t, *J* = 7.1 Hz, 3H), 0.93 (t, *J* = 7.0 Hz, 3H).

Ethyl 3-(nitromethyl)heptanoate (**S10c**): Light pale yellow oil; yield = 85% (118 mg, starting

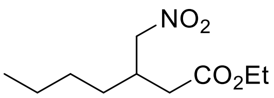

from 100 mg of **S9c**). The ^{1}H NMR data of compound **S10c** match with earlier reported NMR data.[11] ^{1}H NMR (500 MHz, $CDCl_3$) δ 4.54 – 4.41 (m, 2H), 4.15 (q, *J* = 7.1 Hz, 2H), 2.62 (p, *J* = 6.5 Hz, 1H), 2.43 (d, *J* = 6.3 Hz, 2H), 1.42 (q, *J* = 6.7 Hz, 2H), 1.33 (dt, *J* = 7.3, 3.4 Hz, 4H), 1.27 (t, *J* = 7.1 Hz, 3H), 0.90 (t, *J* = 6.8 Hz, 3H).

Ethyl 5,5-dimethyl-3-(nitromethyl)hexanoate (**S10e**): Light pale yellow oil; yield = 63% (85

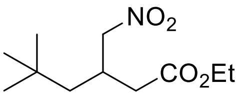

mg, starting from 100 mg of **S9e**). The ^{1}H NMR data of compound **S10e** match with earlier reported NMR data.[11] ^{1}H NMR (500 MHz, $CDCl_3$) δ 4.56 – 4.39 (m, 2H), 4.15 (q, *J* = 7.1 Hz, 2H), 2.65 (p, *J* = 6.0 Hz, 1H), 2.47 (d, *J* = 6.3 Hz, 2H), 1.31 (t, *J* = 4.9 Hz, 2H), 1.27 (t, *J* = 7.1 Hz, 3H), 0.94 (s, 9H).

Ethyl 3-cyclohexyl-4-nitrobutanoate (**S10f**): Light pale yellow oil; yield = 77% (102 mg, starting from 100 mg of **S9f**). The ^{1}H NMR data of compound **S10f** match with earlier reported NMR data.[10] ^{1}H NMR (500 MHz, $CDCl_3$) δ 4.48 (d, J = 6.6 Hz, 2H), 4.15 (q, J = 7.1 Hz, 2H), 2.58 (h, J = 6.5 Hz, 1H), 2.49 (dd, J = 16.3, 5.4 Hz, 1H), 2.34 (dd, J = 16.3, 7.9 Hz, 1H), 1.80 – 1.64 (m, 5H), 1.44 (dddt, J = 11.7, 8.4, 6.2, 2.7 Hz, 1H), 1.26 (t, J = 7.1 Hz, 3H), 1.25 – 1.20 (m, 2H), 1.12 (tt, J = 12.8, 3.3 Hz, 1H), 0.99 (dtt, J = 16.1, 7.7, 3.5 Hz, 2H).

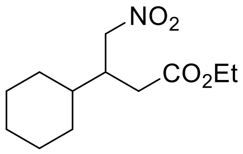

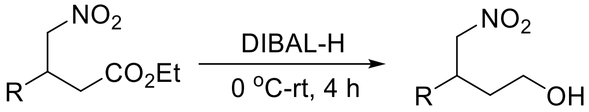

S10 **S11**

To a stirred solution of γ–nitro ester (**S10**) (1.0 equiv.) in 5 mL of dry DCM was added diisobutylaluminum hydride (DIBAL-H) (1.5 equiv., 1.0 M in cyclohexane) dropwise at 0 °C. After the complete addition of DIBAL-H, the mixture was stirred at the same temperature for 3 h and then at room temperature for 1 h. After the completion of starting material (monitored by TLC, $KMnO_4$ staining), the reaction mixture was quenched with saturated aqueous ammonium chloride, and extracted 3 times with DCM. The organic extracts were combined, dried over Na_2SO_4, and evaporated using a rotary evaporator. The crude products were further purified by silica gel column chromatography (using petroleum ether/ethyl acetate from 90:10 to 80:20) as an eluent to give the corresponding alcohols **S11**.

3-(nitromethyl)hexan-1-ol (**S11b**): Light yellow oil; yield = 89% (35 mg, starting from 50 mg of **S10b**). The ^{1}H NMR data of compound **S11b** match with earlier reported NMR data.[12] ^{1}H NMR (500 MHz, $CDCl_3$) δ 4.43 (dd, J = 12.0, 6.6 Hz, 1H), 4.37 (dd, J = 12.0, 6.6 Hz, 1H), 3.79 – 3.65 (m, 2H), 2.43 – 2.32 (m, 1H), 1.76 (s, 1H), 1.73 – 1.54 (m, 2H), 1.43 – 1.31 (m, 4H), 0.91 (ddd, J = 7.0, 4.5, 2.6 Hz, 3H).

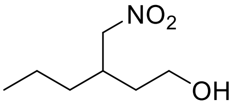

3-(nitromethyl)heptan-1-ol (**S11c**): Light yellow oil; yield = 97% (40 mg, starting from 50 mg of **S10c**). The ^{1}H NMR data of compound **S11c** match with earlier reported NMR data.[13] ^{1}H NMR (500 MHz, $CDCl_3$) δ 4.48 – 4.33 (m, 2H), 3.78 – 3.64 (m, 2H), 2.35 (p, J = 6.5 Hz, 1H), 1.84 (s, 1H), 1.71 – 1.54 (m, 2H), 1.43 – 1.26 (m, 6H), 0.93 – 0.83 (m, 3H).

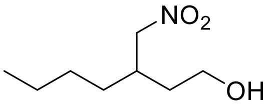

5,5-dimethyl-3-(nitromethyl)hexan-1-ol (**S11e**): Light yellow oil; yield = 97% (40 mg, starting from 50 mg of **S10e**). ^{1}H NMR (500 MHz, $CDCl_3$) δ 4.44 – 4.35 (m, 2H), 3.73 (dtd, *J* = 19.6, 10.7, 4.6 Hz, 2H), 2.40 (dt, *J* = 11.6, 5.3 Hz, 1H), 1.73 – 1.63 (m, 2H), 1.40 (t, *J* = 4.5 Hz, 1H), 1.28 (dd, *J* = 4.8, 3.7 Hz, 2H), 0.93 (s, 9H). ^{13}C NMR (126 MHz, $CDCl_3$) δ 81.03, 60.25, 45.21, 36.74, 31.40, 31.14, 29.67. HRMS (ESI+): calcd. for $C_9H_{20}NO_3$ [M+H]+: 190.1443, found: 190.1443.

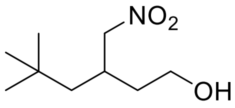

3-cyclohexyl-4-nitrobutan-1-ol (**S11f**): Light yellow oil; yield = 83% (34 mg, starting from 50 mg of **S10f**). ^{1}H NMR (500 MHz, $CDCl_3$) δ 4.41 (qd, *J* = 12.3, 6.9 Hz, 2H), 3.70 (tq, *J* = 7.7, 4.2 Hz, 2H), 2.28 (h, *J* = 7.2 Hz, 1H), 1.75 (dq, *J* = 11.8, 6.4 Hz, 3H), 1.70 – 1.60 (m, 3H), 1.50 (td, *J* = 14.3, 6.4 Hz, 1H), 1.42 (dtd, *J* = 14.6, 7.1, 3.3 Hz, 1H), 1.28 – 1.17 (m, 3H), 1.11 (ddt, *J* = 25.5, 12.6, 3.4 Hz, 1H), 1.01 (ddd, *J* = 15.3, 12.4, 3.1 Hz, 2H). ^{13}C NMR (126 MHz, $CDCl_3$) δ 78.02, 60.95, 40.09, 39.42, 31.67, 29.59, 29.49, 26.58, 26.53, 26.50. HRMS (ESI+): calcd. for $C_{10}H_{20}NO_3$ [M+H]+: 202.1443, found: 202.1443.

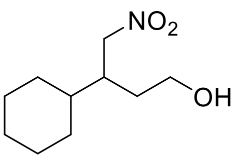

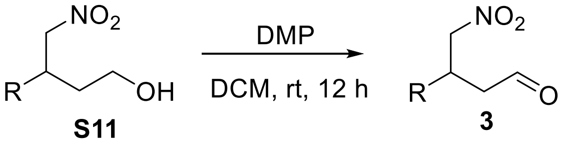

To a stirred solution of (**S11**) (1.0 equiv.) in 5 mL of dry DCM was added Dess–Martin periodinane (DMP) (1.16 equiv.) at 0 °C. After the complete addition of DMP, the mixture was stirred at room temperature for 12 h. After the completion of starting material (monitored by TLC, $KMnO_4$ staining), the reaction mixture was extracted 3 times with DCM. The organic extracts were combined, dried over Na_2SO_4, and evaporated using a rotary evaporator. The crude products were further purified by silica gel column chromatography (using petroleum ether/ethyl acetate from 90:10 to 80:20) as an eluent to give the corresponding γ–nitroaldehydes **3**.

3-(nitromethyl)hexanal (**3b**): Clear oil; yield = 83% (28 mg, starting from 35 mg of **S11b**). The ^{1}H NMR data of compound **3b** match with earlier reported NMR data.[14] ^{1}H NMR (500 MHz, $CDCl_3$) δ 9.79 – 9.78 (s, 1H), 4.50 – 4.39 (m, 2H), 2.77 – 2.55 (m, 3H), 1.38 (dtq, *J* = 15.5, 6.2, 3.1 Hz, 4H), 0.93 (t, *J* = 7.0 Hz, 3H).

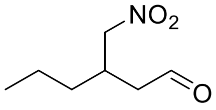

3-(nitromethyl)heptanal (**3c**): Clear oil; yield = 79% (31 mg, starting from 40 mg of **S11c**). The

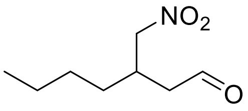

^{1}H NMR data of compound **3c** match with earlier reported NMR data.[14] ^{1}H NMR (500 MHz, $CDCl_3$) δ 9.78 (s, 1H), 4.45 (dd, *J* = 6.0, 1.6 Hz, 2H), 2.76 – 2.59 (m, 3H), 1.43 (dd, *J* = 8.5, 5.5 Hz, 2H), 1.32 (dd, *J* = 7.0, 3.4 Hz, 4H), 0.92 – 0.88 (m, 3H).

5,5-dimethyl-3-(nitromethyl)hexanal (**3e**): Clear oil; yield = 73% (29 mg, starting from 40 mg

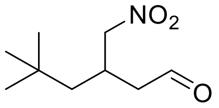

of **S11e**). ^{1}H NMR (500 MHz, $CDCl_3$) δ 9.74 (s, 1H), 4.43 (qd, *J* = 12.1, 6.0 Hz, 2H), 2.77 – 2.58 (m, 3H), 1.35 (dd, *J* = 14.7, 4.9 Hz, 1H), 1.25 (dd, *J* = 14.7, 4.8 Hz, 1H), 0.93 (s, 9H). ^{13}C NMR (126 MHz, $CDCl_3$) δ 200.12, 79.88, 47.64, 45.03, 31.21, 29.61, 28.74. HRMS (ESI+): calcd. for $C_9H_{18}NO_3$ [M+H]+: 188.1287, found: 188.1287.

3-cyclohexyl-4-nitrobutanal (**3f**): Clear oil; yield = 88% (13 mg, starting from 15 mg of **S11f**).

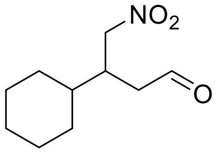

The ^{1}H NMR data of compound **3f** match with earlier reported NMR data.[15] ^{1}H NMR (500 MHz, $CDCl_3$) δ 9.78 (s, 1H), 4.52 – 4.35 (m, 2H), 2.72 – 2.62 (m, 2H), 2.59 – 2.48 (m, 1H), 1.80 – 1.63 (m, 5H), 1.42 (ddt, *J* = 11.9, 5.0, 3.2 Hz, 1H), 1.27 – 1.17 (m, 2H), 1.12 (tt, *J* = 12.8, 3.3 Hz, 1H), 0.98 (dtt, *J* = 16.1, 7.7, 3.7 Hz, 2H).

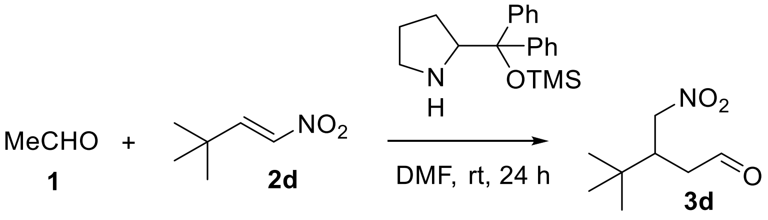

4,4-dimethyl-3-(nitromethyl)pentanal (**3d**): Racemic 4,4-dimethyl-3-(nitromethyl)pentanal **3d**

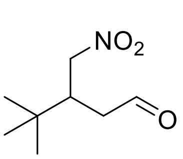

was prepared according to previously described procedures.[15] Briefly, 500 µl of DMF, 760 µL of 2-propanol and 250 µl of a 0.8 M solution of racemic α,α-diphenyl-2-pyrrolidinemethanol trimethylsilyl ether in DMF were added to (*E*)-3,3-dimethyl-1-nitrobut-1-ene **2d** (1 mmol) in a vial under nitrogen at room temperature. 500 µl of a 10 M acetaldehyde (**1**) solution in anhydrous DMF cooled to 4 °C was added dropwise. After stirring for 24 h, the reaction mixture was quenched with 1 N HCl and extracted twice with ethyl acetate. The organic layers were dried over anhydrous $MgSO_4$, filtered, and concentrated *in vacuo*. Purification by column chromatography gave 4,4-dimethyl-3-(nitromethyl)pentanal **3d (**12.1 mg, 7 % yield). The ^{1}H NMR data of compound **3d** match with

earlier reported NMR data.[15] ^{1}H NMR (500 MHz, $CDCl_3$) δ 9.77 (s, J = 1.4 Hz, 1H), 4.56 (dd, J = 12.6, 4.3 Hz, 1H), 4.25 (dd, J = 12.6, 8.7 Hz, 1H), 2.81 – 2.75 (m, 1H), 2.74 – 2.68 (m, 1H), 2.54 – 2.46 (m, 1H), 0.95 (s, 9H).

Expression and purification of 4-OT

The expression and purification of 4-OT mutants were performed using slightly modified previously reported procedures.[16] Briefly, 1 l of LB medium substituted with 0.5 % glycerol and 100 µg/ml ampicillin was inoculated from a glycerol stock with *E. coli* BL21(DE3) harboring a pJexpress 414 vector with the respective 4-OT gene. After 1 h at 37 °C, 200 rpm the expression was induced with 100 µM IPTG and continued at 37 °C, 200 rpm overnight. After the expression of 4-OT the cells were collected and the enzymes were purified according to previously reported methods.[17,18] The enzyme concentration was determined using the Waddell method.[19] The purified enzymes were flash frozen in liquid nitrogen and stored at -20 °C until further used. For each purified 4-OT variant the removal of the N-terminal methionine was confirmed by analysis of the protein mass using electron spray ionization mass spectrometry.

Enzymatic semi-preparative scale reactions for **3b-f**

3-(nitromethyl)hexanal **3b**

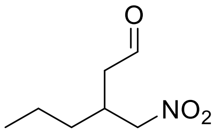

The 4-OT catalyzed addition of acetaldehyde **1** to (*E*)-1-nitropent-1-ene **2b** was performed with 150 mM **1**, 3 mM **2b**, 100 µM 4-OT L8Y/M45Y/F50A or 120 µM 4-OT A33D and 15 % ethanol in 20 mM NaH_2PO_4 pH 6.5. The total volume of the reaction was 43.4 ml. The reaction was followed by UV-vis spectroscopy until completion of the reaction was observed (4-OT L8Y/M45Y/F50A: 30 min, 4-OT A33D: 35 min). The solution was saturated with NaCl and the product extracted with 3 x 40 mL toluene. The organic layers were combined, dried over anhydrous Na_2SO_4, and concentrated *in vacuo* to give 3-(nitromethyl)hexanal **3b** (4-OT L8Y/M45Y/F50A: 12.7 mg, 61 % yield, 4-OT A33D: 14.0 mg, 68 % yield). The ^{1}H NMR data of **3b** were in accordance with previously reported NMR data.[14] ^{1}H NMR (500 MHz, $CDCl_3$) δ 9.78 (s, 1H), 4.48 – 4.41 (m, 2H), 2.77 – 2.69 (m, 1H), 2.69 – 2.55 (m, 2H), 1.42 – 1.33 (m, 4H), 0.92 (t, 3H). The enantiomeric ratio was determined by GC analysis using a chiral-phase G-TA column (130 °C isocratic, 1.97 ml/min). Flame ionization detection t_R: **3b** = 21.2 min and **3b** = 23.6 min, resulted in the following enantiomeric ratios: racemic **3b** = 50:50, 4-OT L8Y/M45Y/F50A **3b** = 98:2 and 4-OT A33D **3b** = 2:98.

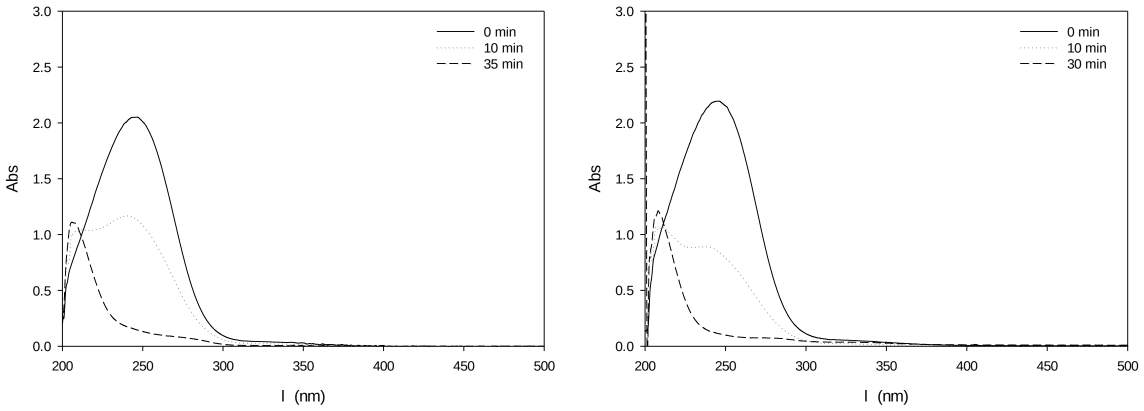

Supplementary Figure 1: UV spectra monitoring the addition of **1** to **2b** catalyzed by 4-OT A33D (left) and 4-OT L8Y/M45Y/F50A (right).

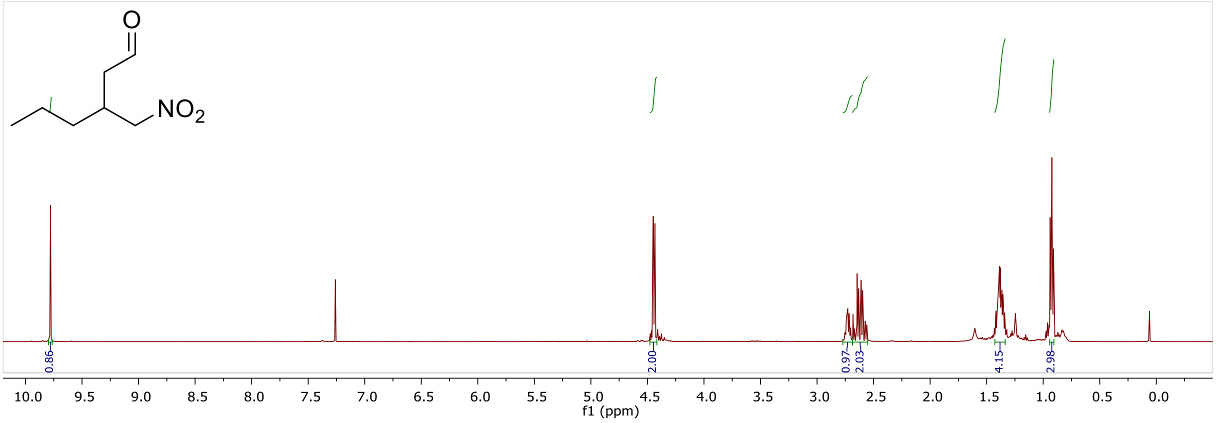

Supplementary Figure 2: ^{1}H NMR spectrum of 3-(nitromethyl)hexanal (**3b**) synthesized with 4-OT L8Y/M45Y/F50A.

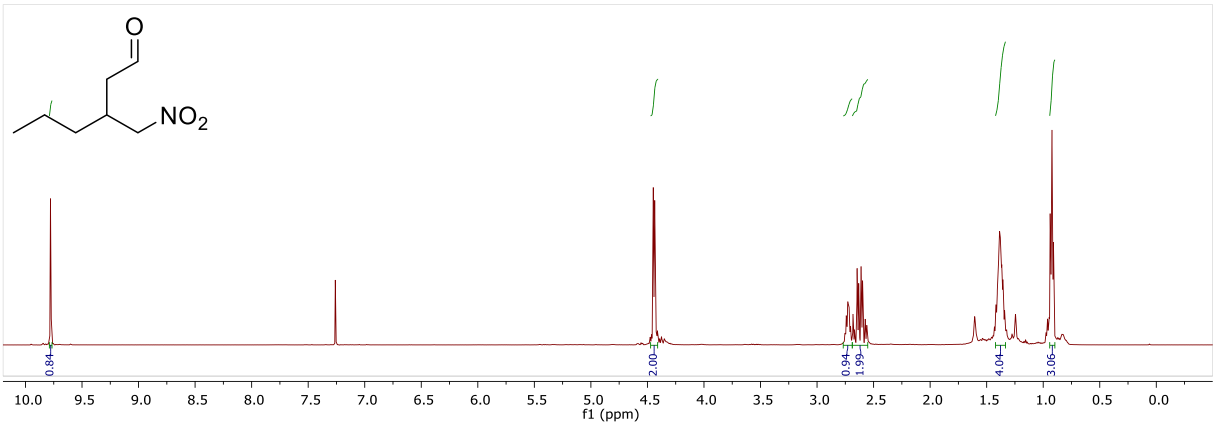

Supplementary Figure 3: ^{1}H NMR spectrum 3-(nitromethyl)hexanal (**3b**) synthesized with 4-OT A33D.

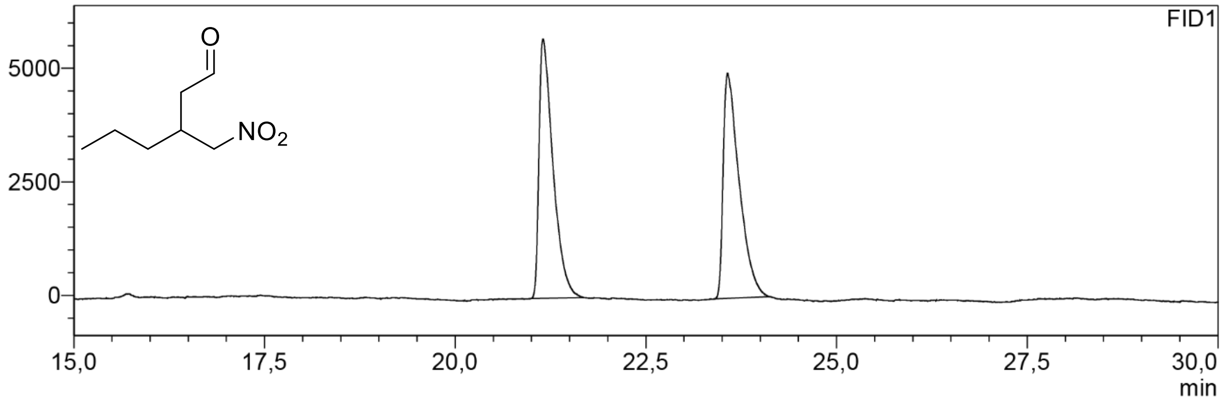

Supplementary Figure 4: GC chromatogram of racemic **3b**.

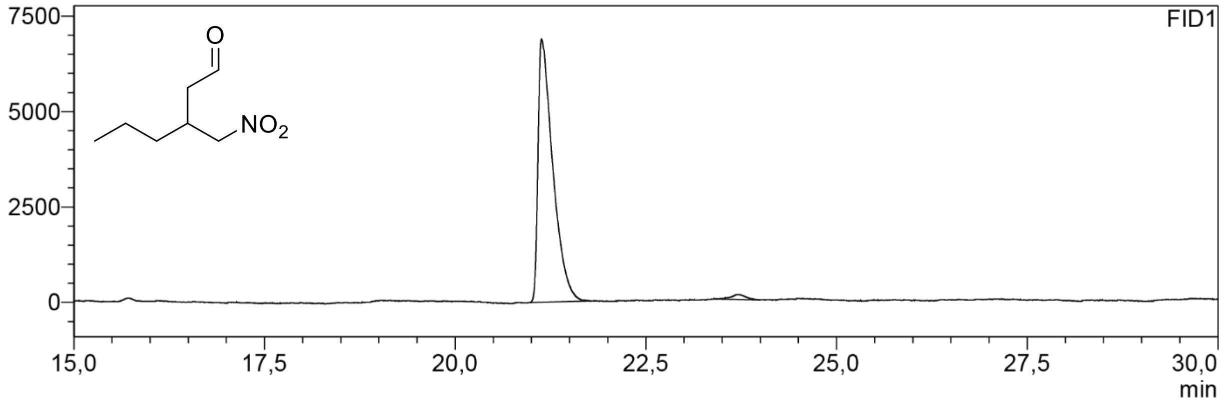

Supplementary Figure 5: GC chromatogram of **3b** obtained with 4-OT L8Y/M45Y/F50A.

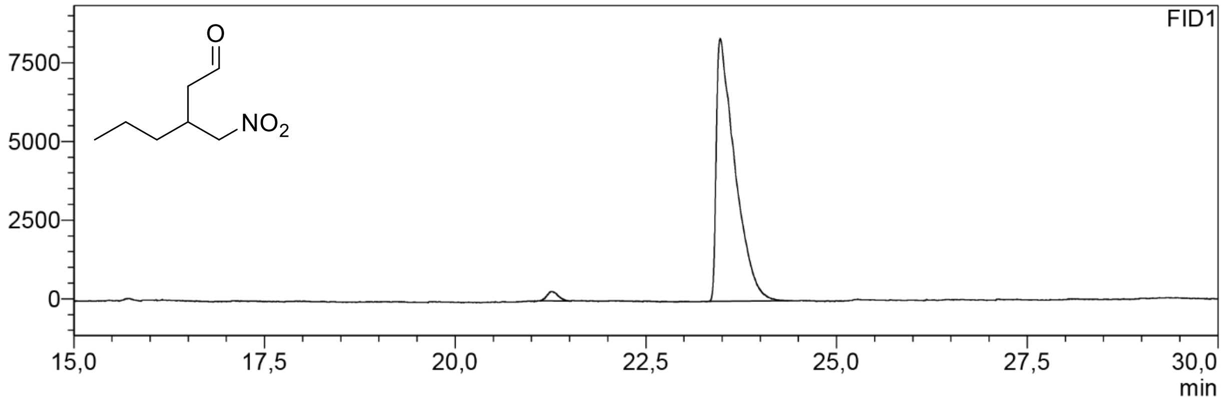

Supplementary Figure 6: GC chromatogram of **3b** obtained with 4-OT A33D.

3-(nitromethyl)heptanal **3c**

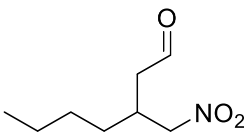

The 4-OT catalysed addition of **1** to (*E*)-1-nitrohex-1-ene **2c** was performed with 150 mM **1**, 3 mM **2c**, 100 µM 4-OT and 15 % ethanol in 20 mM NaH_2PO_4 pH 6.5. The total volume of the reaction was 38.7 ml. The reaction was followed by UV-vis spectroscopy until completion of the reaction was observed (4-OT L8Y/M45Y/F50A: 30 min, 4-OT A33D: 30 min). The solution was saturated with NaCl and the product extracted with 3 x 40 mL toluene. The organic layers were combined, dried over anhydrous Na_2SO_4, and concentrated *in vacuo* to give 3-(nitromethyl)heptanal **3c** (4-OT L8Y/M45Y/F50A: 15.0 mg, 75 % yield, 4-OT A33D: 18.4 mg, 92 % yield). The ^{1}H NMR data of **3c** were in accordance with previously reported NMR data.[14,20] ^{1}H NMR (500 MHz, $CDCl_3$) δ 9.78 (s, 1H), 4.44 (dd, J = 6.0, 1.7 Hz, 2H), 2.75 – 2.67 (m, 1H), 2.66 – 2.56 (m, 2H), 1.45 – 1.39 (m, 2H), 1.34 – 1.29 (m, 4H), 0.90 (m, 3H). The enantiomeric ratio was determined by GC analysis using a chiral-phase G-TA column (130 °C isocratic, 1.97 ml/min). Flame ionization detection t_R: (*S*)-**3c** = 25.8 min and (*R*)-**3c** = 28.6 min, resulted in the following enantiomeric ratios (*S*:*R*): racemic **3c** = 50:50, 4-OT L8Y/M45Y/F50A **3c** = > 99:1 and 4-OT A33D **3c** = 1:99. The assignment of the absolute configuration was based on earlier reported chiral-phase GC data.[20]

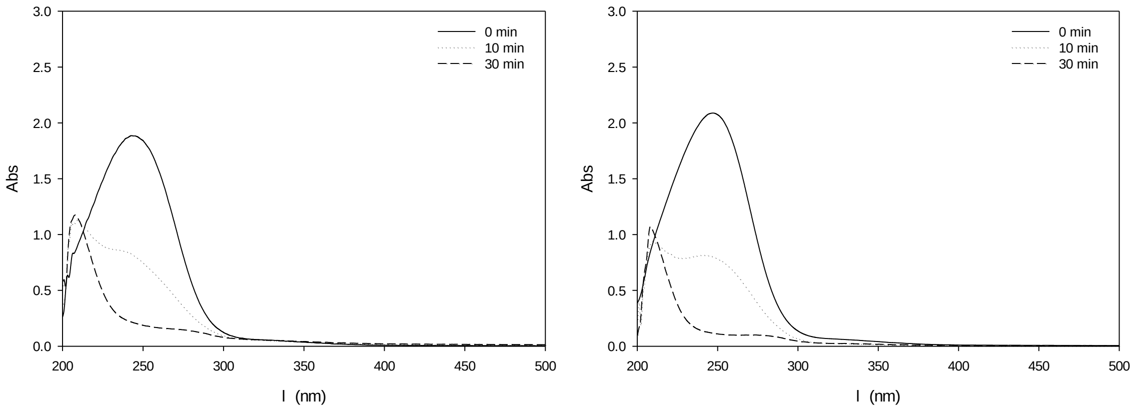

Supplementary Figure 7: UV spectra monitoring the addition of **1** to **2c** catalyzed by 4-OT A33D (left) and 4-OT L8Y/M45Y/F50A (right).

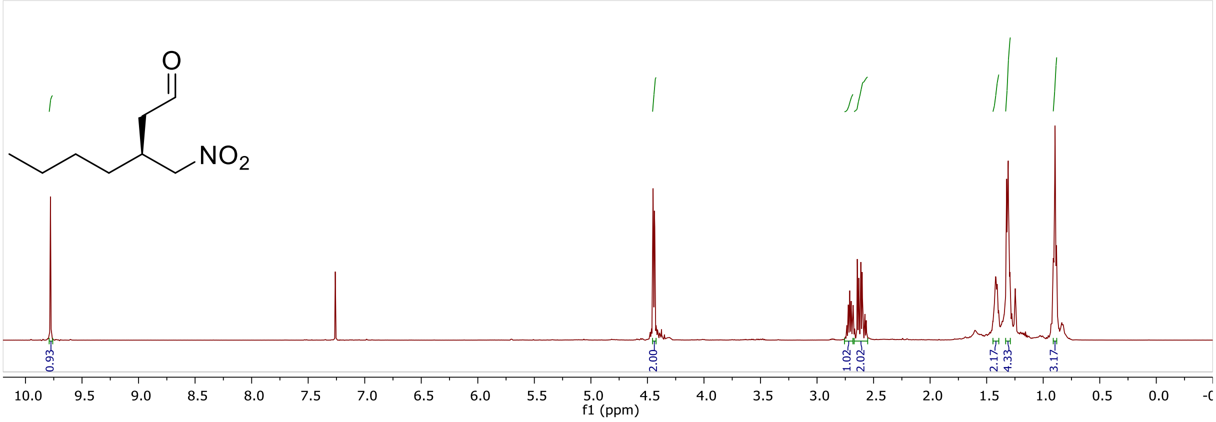

Supplementary Figure 8: ^{1}H NMR spectrum of (*S*)-3-(nitromethyl)heptanal (**3c**) synthesized with 4-OT L8Y/M45Y/F50A.

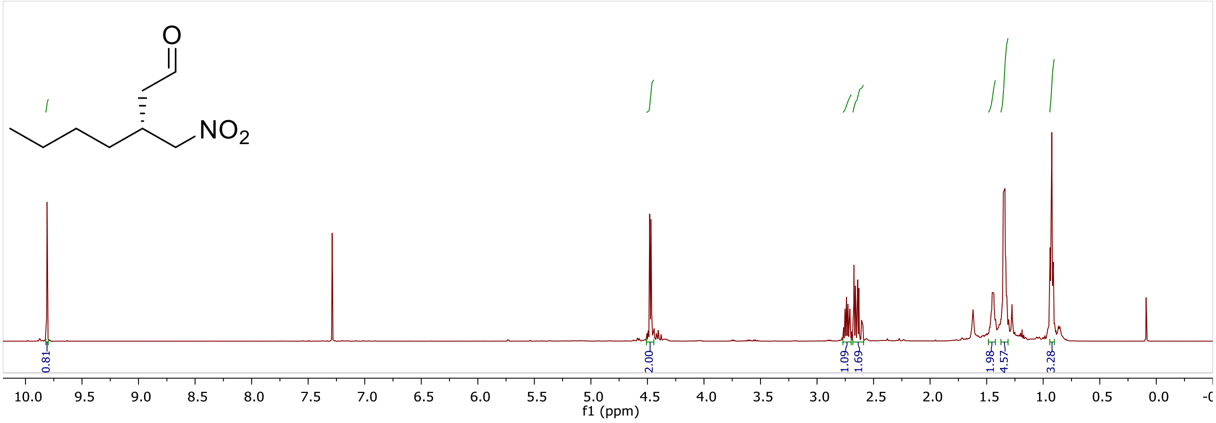

Supplementary Figure 9: ^{1}H NMR spectrum of (*R*)-3-(nitromethyl)heptanal (**3c**) synthesized with 4-OT A33D.

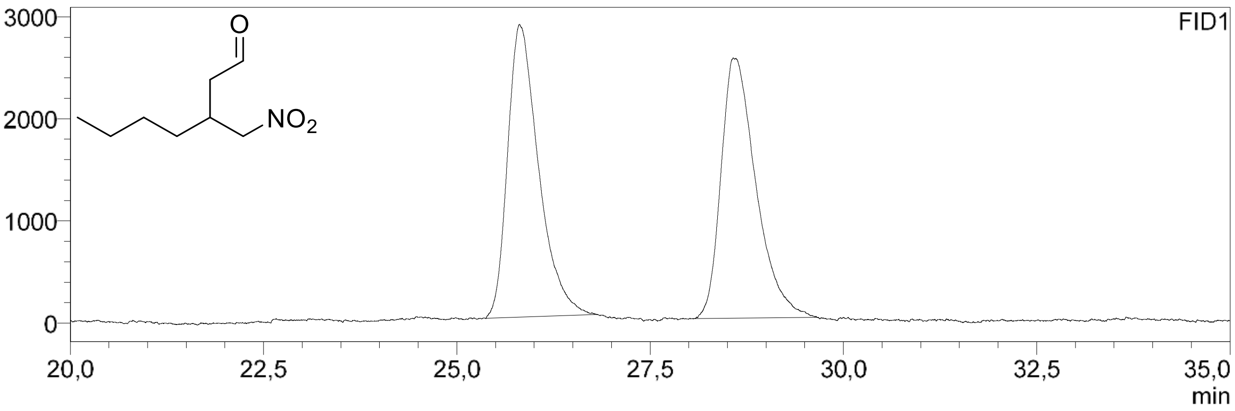

Supplementary Figure 10: GC chromatogram of racemic **3c**.

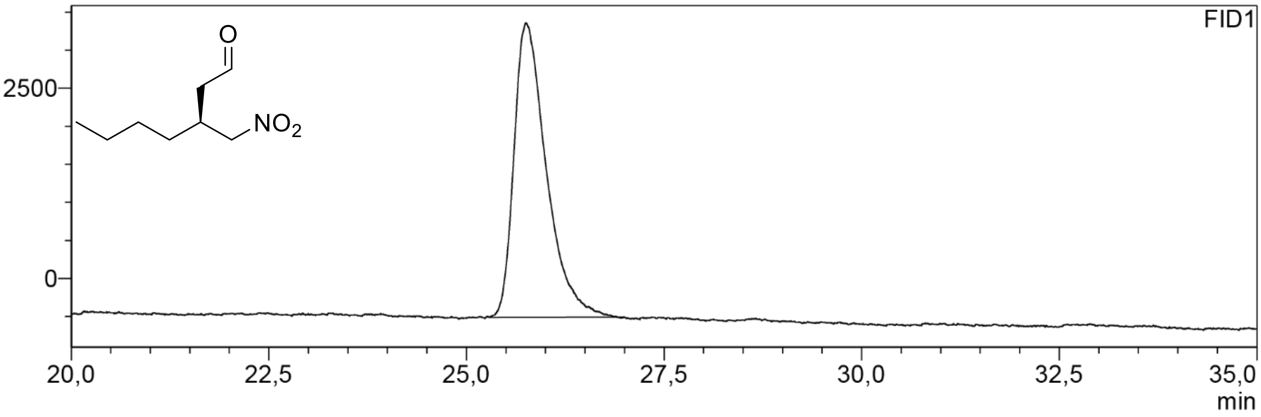

Supplementary Figure 11: GC chromatogram of (*S*)-**3c** obtained with 4-OT L8Y/M45Y/F50A.

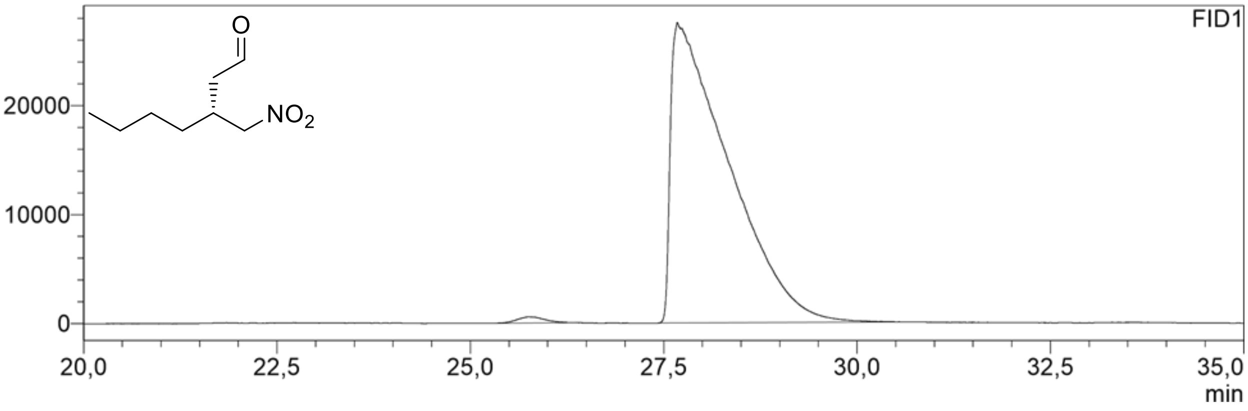

Supplementary Figure 12: GC chromatogram of (*R*)-**3c** obtained with 4-OT A33D.

4,4-dimethyl-3-(nitromethyl)pentanal **3d**

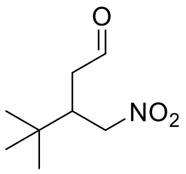

The 4-OT catalyzed addition of **1** to (*E*)-3,3-dimethyl-1-nitrobut-1-ene **2d** was performed with 150 mM **1**, 3 mM **2d**, 100 µM 4-OT and 15 % ethanol in 20 mM NaH_2PO_4 pH 6.5. The total volume of the reaction was 38.7 ml. The reaction was followed by UV-vis spectroscopy until completion of the reaction was observed (4-OT L8Y/M45Y/F50A: 150 min, 4-OT A33D: 150 min). The solution was saturated with NaCl and the product extracted with 3 x 30 mL toluene. The organic layers were combined, dried over anhydrous Na_2SO_4, and concentrated *in vacuo* to give 4,4-dimethyl-3-(nitromethyl)pentanal **3d** (4-OT L8Y/M45Y/F50A: 9.3 mg, 46 % yield, 4-OT A33D: 12.2 mg, 61 % yield). The ^{1}H NMR data of **3d** were in accordance with previously reported literature.[15] ^{1}H NMR (500 MHz, $CDCl_3$) δ 9.76 (s, 1H), 4.56 (dd, J = 12.6, 0.8 Hz, 1H), 4.25 (dd, J = 8.7, 0.8 Hz, 1H), 2.81 – 2.74 (m, 1H), 2.74 – 2.66 (m, 1H), 2.53 – 2.46 (m, 1H), 0.95 (s, 9H). The enantiomeric ratio was determined by GC analysis using a chiral-phase G-TA column (130 °C isocratic, 1.97 ml/min). Flame ionization detection t_R: **3d** = 26.3 min and **3d** = 33.4 min, resulted in the following enantiomeric ratios: racemic **3d** = 43:57, 4-OT L8Y/M45Y/F50A **3d** = 98:2 and 4-OT A33D **3d** = > 1:99.

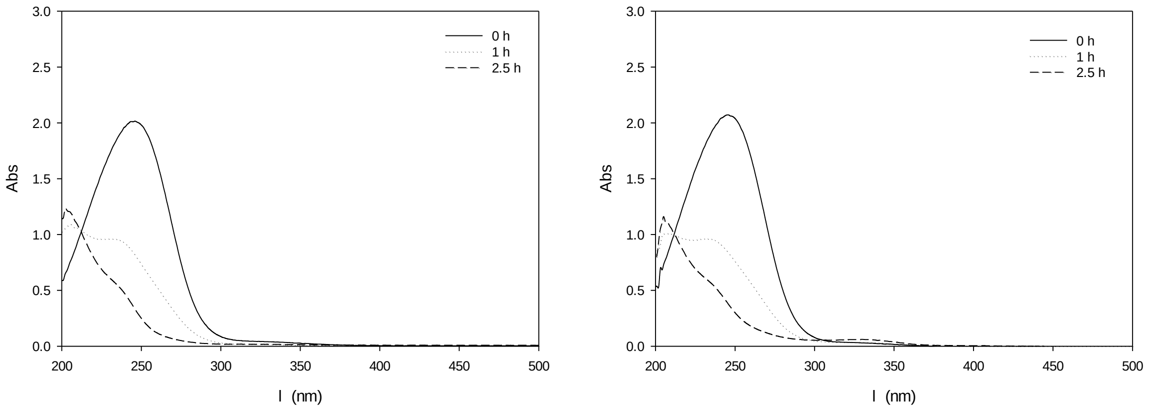

Supplementary Figure 13: UV spectra monitoring the addition of **1** to **2d** catalyzed by 4-OT A33D (left) and 4-OT L8Y/M45Y/F50A (right).

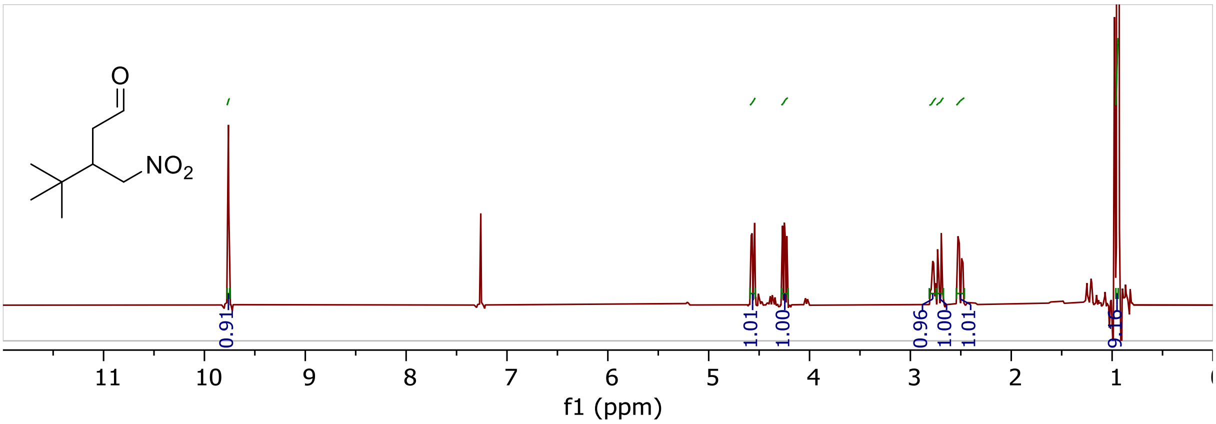

Supplementary Figure 14: ^{1}H NMR spectrum of 4,4-dimethyl-3-(nitromethyl)pentanal (**3d**) synthesized with 4-OT L8Y/M45Y/F50A. Minor peaks (4 – 5 ppm) are caused by the non-enzymatic hydration of **2d**.

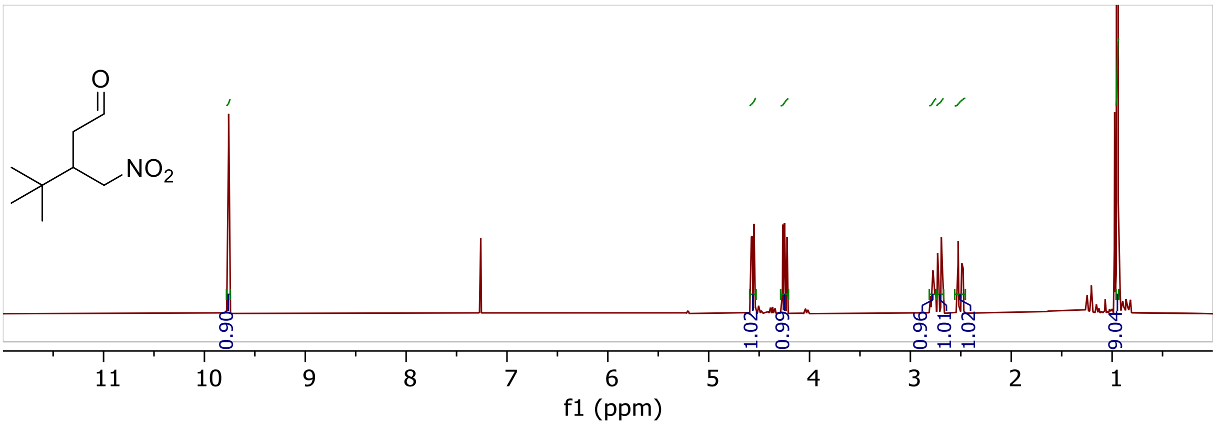

Supplementary Figure 15: ^{1}H NMR spectrum of 4,4-dimethyl-3-(nitromethyl)pentanal (**3d**) synthesized with 4-OT A33D. Minor peaks (4 – 5 ppm) are caused by the non-enzymatic hydration of **2d**.

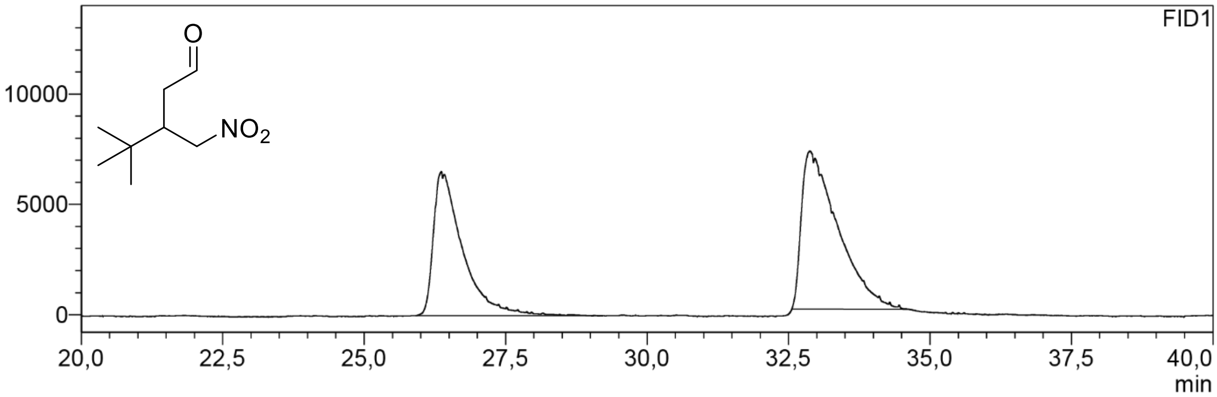

Supplementary Figure 16: GC chromatogram of racemic **3d**.

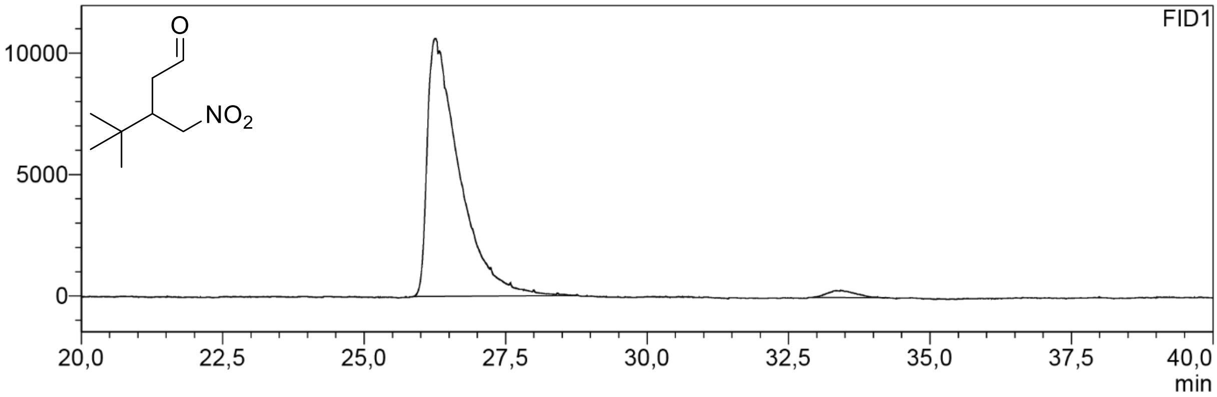

Supplementary Figure 17: GC chromatogram of **3d** obtained with 4-OT L8Y/M45Y/F50A.

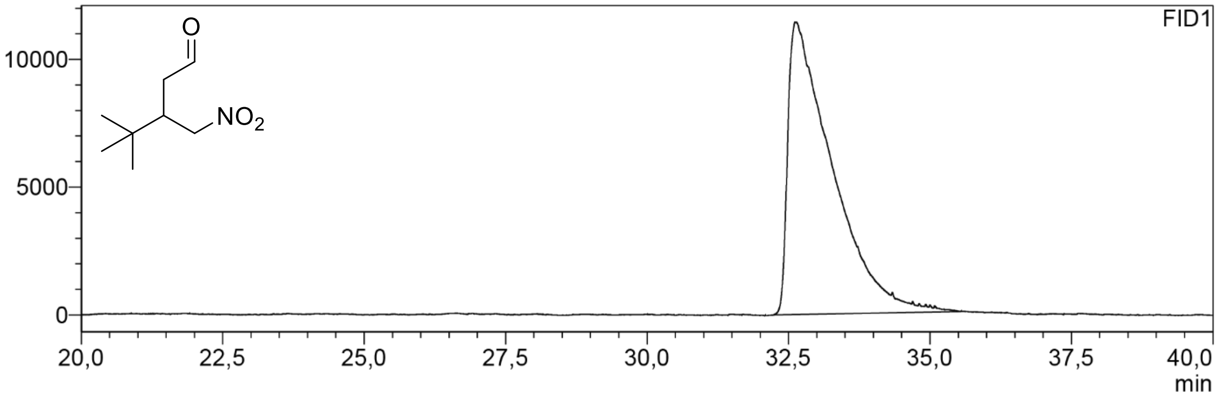

Supplementary Figure 18: GC chromatogram of **3d** obtained with 4-OT A33D.

5,5-dimethyl-3-(nitromethyl)hexanal **3e**

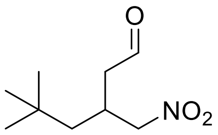

The 4-OT catalyzed addition of **1** to (*E*)-4,4-dimethyl-1-nitropent-1-ene **2e** was performed with 150 mM **1**, 3 mM **2e**, 100 µM 4-OT and 15 % ethanol (10 % ethanol for 4-OT L8Y/M45Y/F50A) in 20 mM NaH_2PO_4 pH 6.5. The total volume of the reaction was 34.9 ml. The reaction was followed by UV-vis spectroscopy until completion of the reaction was observed (4-OT L8Y/M45Y/F50A: 20 min, 4-OT A33D: 45 min). The solution was saturated with NaCl and the product extracted with 3 x 30 mL toluene. The organic layers were combined, dried over anhydrous Na_2SO_4, and concentrated *in vacuo* to give 5,5-dimethyl-3-(nitromethyl)hexanal **3e** (4-OT L8Y/M45Y/F50A: 14.6 mg, 74 % yield, 4-OT A33D: 17.3 mg, 88 % yield). ^{1}H NMR (500 MHz, $CDCl_3$) δ 9.75 (s, 1H), δ 4.44 (qd, *J* = 12.1, 5.9 Hz, 2H), 2.79 – 2.69 (m, 2H), 2.67 – 2.60 (m, 1H), 1.36 (dd, *J* = 14.6, 4.9 Hz, 1H), 1.29 – 1.23 (m, 1H), 0.94 (s, 9H). ^{13}C NMR (126 MHz, $CDCl_3$) δ 200.12, 79.90, 47.67, 45.06, 31.24, 29.64, 28.78. HRMS (ESI+): calcd. for $C_9H_{18}NO_3$ [M+H]+: 188.12812, found: 188.12775 (4-OT L8Y/M45Y/F50A) and 188.12778 (4-OT A33D). The enantiomeric ratio was determined by GC analysis using a chiral-phase G-TA column (130 °C isocratic, 1.97 ml/min). Flame ionization detection t_R: **3e** = 20.5 min and **3e** = 21.9 min, resulted in the following enantiomeric ratios: racemic **3e** = 50:50, 4-OT L8Y/M45Y/F50A **3e** = > 99:1 and 4-OT A33D **3c** = < 1:99.

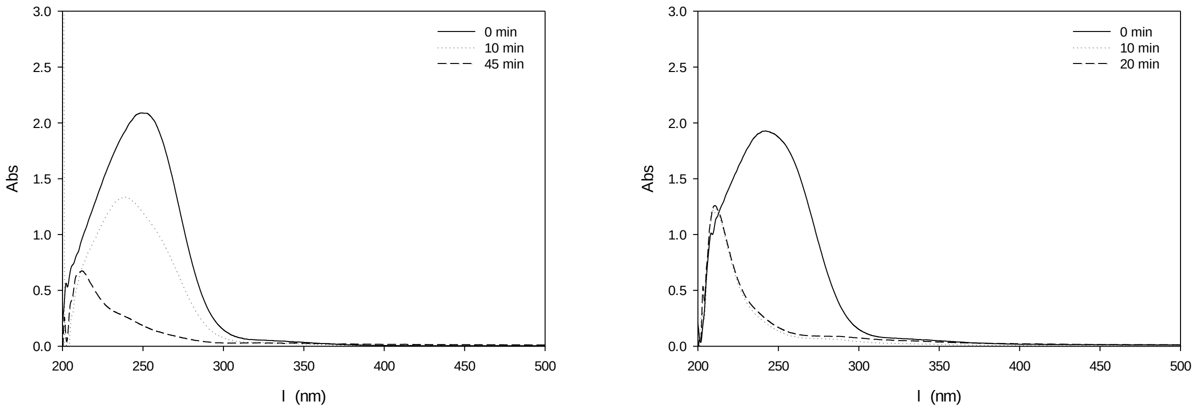

Supplementary Figure 19: UV spectra monitoring the addition of **1** to **2e** catalyzed by 4-OT A33D (left) and 4-OT L8Y/M45Y/F50A (right).

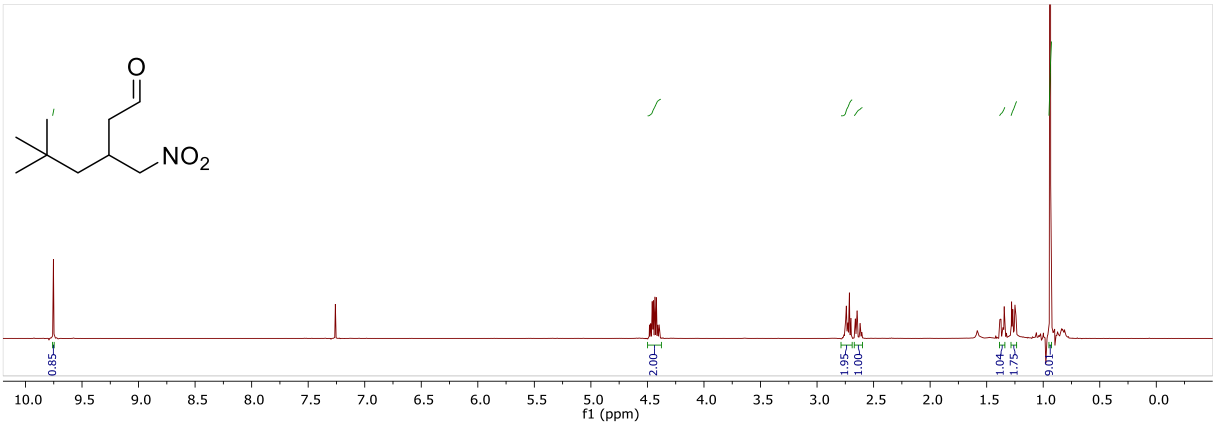

Supplementary Figure 20: ^{1}H NMR spectrum of 5,5-dimethyl-3-(nitromethyl)hexanal (**3e**) synthesized with 4-OT L8Y/M45Y/F50A.

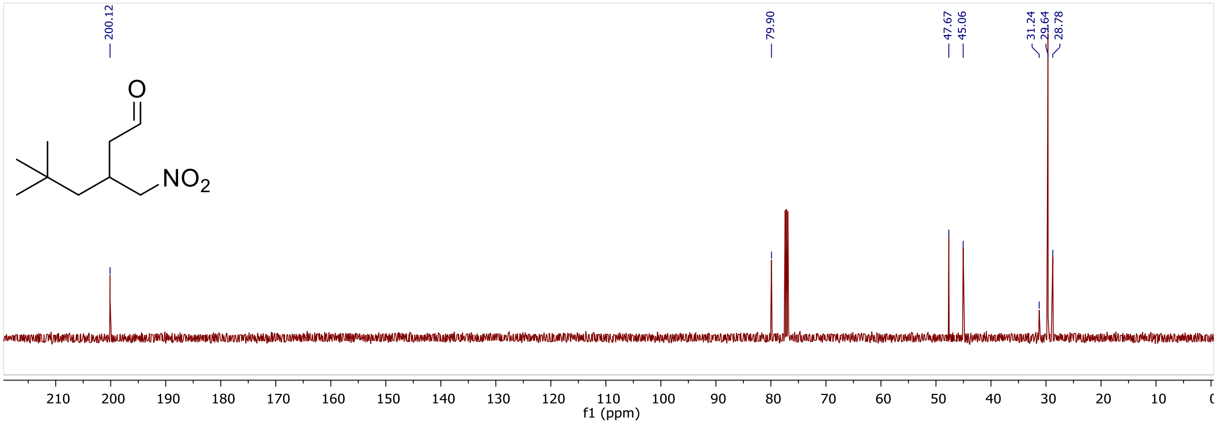

Supplementary Figure 21: ^{13}C NMR spectrum of 5,5-dimethyl-3-(nitromethyl)hexanal (**3e**) synthesized with 4-OT L8Y/M45Y/F50A.

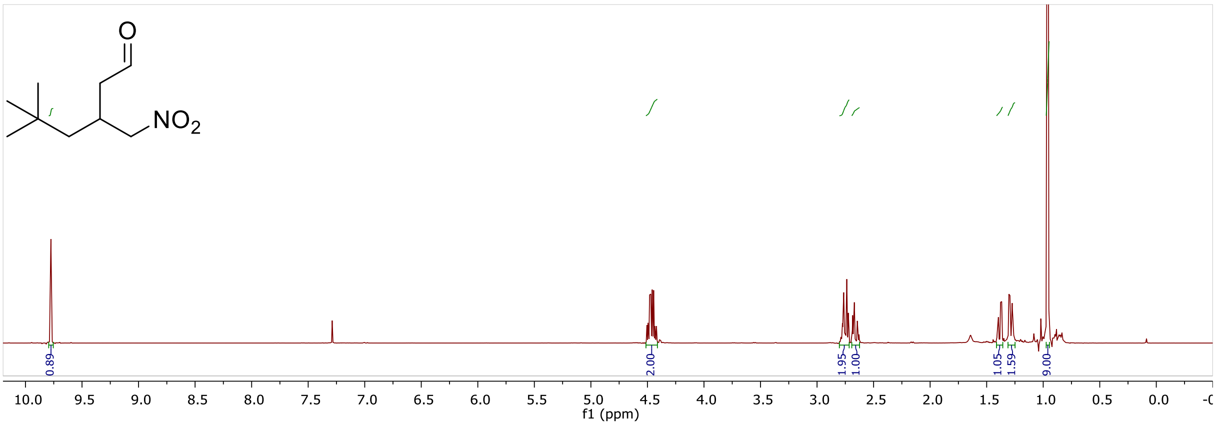

Supplementary Figure 22: ^{1}H NMR spectrum of 5,5-dimethyl-3-(nitromethyl)hexanal (**3e**) synthesized with 4-OT A33D.

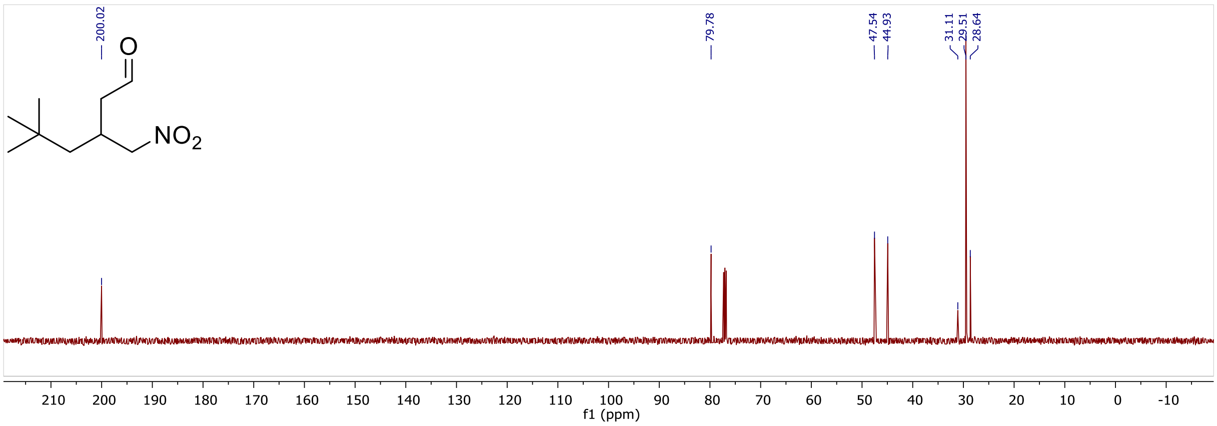

Supplementary Figure 23: ^{13}C NMR spectrum of 5,5-dimethyl-3-(nitromethyl)hexanal (**3e**) synthesized with 4-OT A33D.

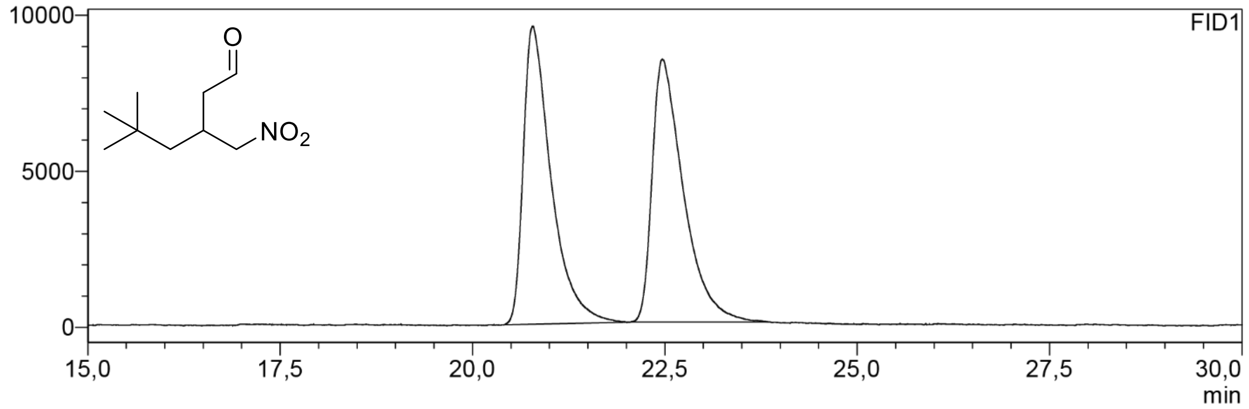

Supplementary Figure 24: GC chromatogram of racemic **3e**.

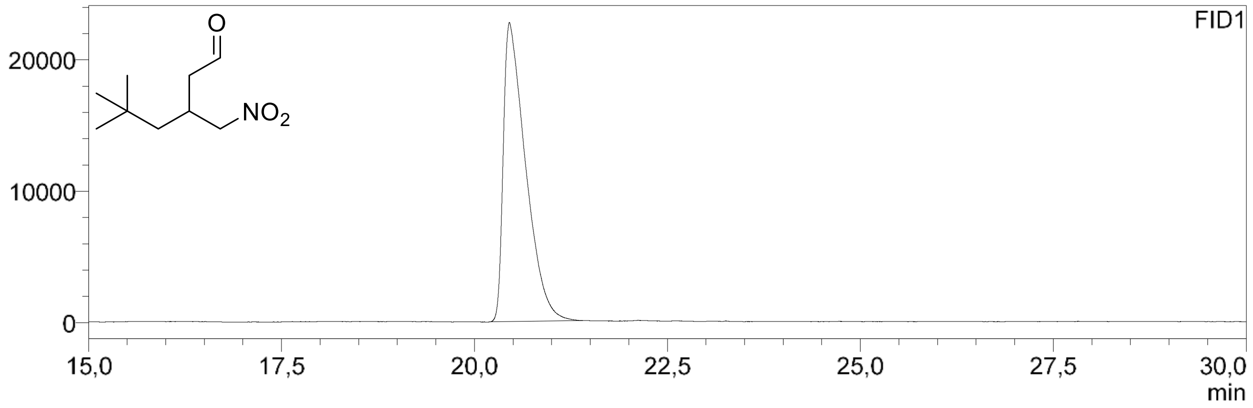

Supplementary Figure 25: GC chromatogram of **3e** obtained with 4-OT L8Y/M45Y/F50A.

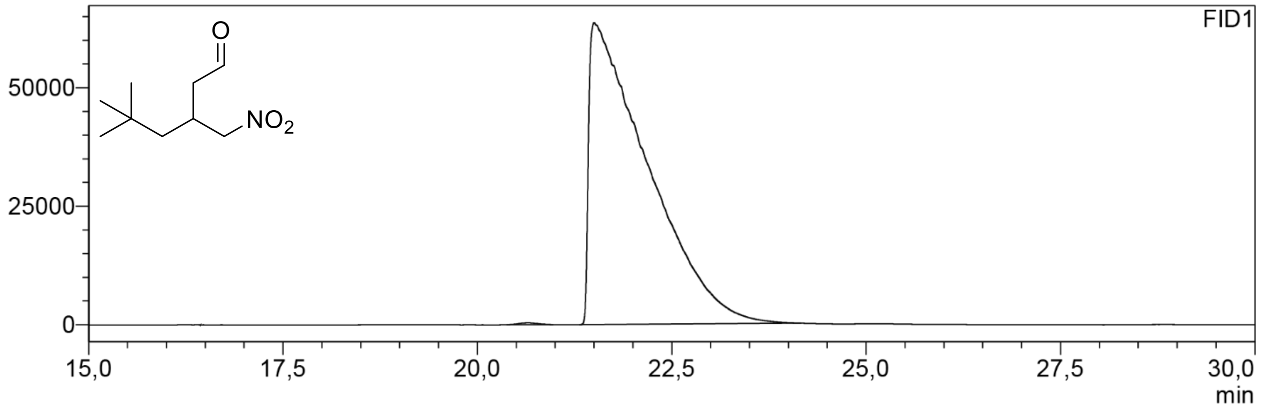

Supplementary Figure 26: GC chromatogram of **3e** obtained with 4-OT A33D.

3-cyclohexyl-4-nitrobutanal **3f**

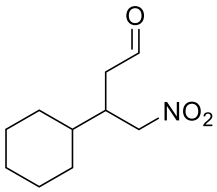

The 4-OT catalyzed addition of **1** to (*E*)-(2-nitrovinyl)cyclohexane **2f** was performed with 150 mM **1**, 3 mM **2f**, 100 µM 4-OT and 15 % ethanol in 20 mM NaH_2PO_4 pH 6.5. The total volume of the reaction was 32.2 ml. The reaction was followed by UV-vis spectroscopy until completion of the reaction was observed (4-OT L8Y/M45Y/F50A: 20 min, 4-OT A33D: 25 min). The solution was saturated with NaCl and the product extracted with 3 x 30 mL toluene. The organic layers were combined, dried over anhydrous Na_2SO_4, and concentrated *in vacuo* to give 3-cyclohexyl-4-nitrobutanal **3f** (4-OT L8Y/M45Y/F50A: 17.8 mg, 92 % yield). Silica gel purification gave 3-cyclohexyl-4-nitrobutanal **3f** (4-OT A33D: 12.1 mg, 63 % yield). The ^{1}H NMR data were in accordance with previously reported NMR data.[15] ^{1}H NMR (500 MHz, $CDCl_3$) δ 9.78 (s, 1H), 4.47 (dd, J = 12.4, 5.7 Hz, 1H), 4.40 (dd, J = 12.4, 7.3 Hz, 1H), 2.72 – 2.62 (m, 2H), 2.58 – 2.51 (m, 1H), 1.80 – 1.64 (m, 5H), 1.47 – 1.38 (m, 1H), 1.27 – 0.92 (m, 5H). The enantiomeric ratio was determined by GC analysis using a chiral-phase G-TA column (150 °C isocratic, 2.14 ml/min). Flame ionization detection t_R: (*R*)-**3f** = 34.5 min and (*S*)-**3f** = 37.5 min, resulted in the following enantiomeric ratios (*R*:*S*): racemic **3f** = 50:50, 4-OT L8Y/M45Y/F50A **3f** = > 99:1 and 4-OT A33D **3f** = < 1:99. The assignment of the absolute configuration was based on earlier reported chiral-phase GC data.[15]

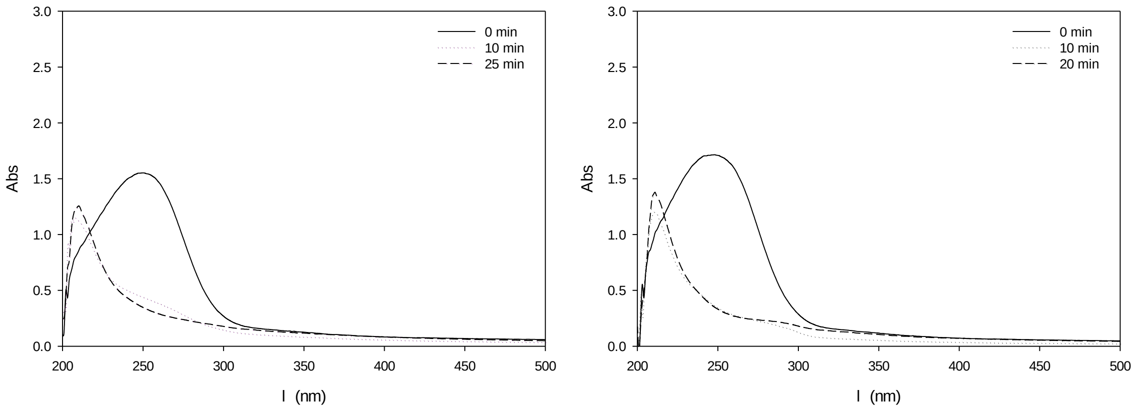

Supplementary Figure 27: UV spectra monitoring the addition of **1** to **2f** catalyzed by 4-OT A33D (left) and 4-OT L8Y/M45Y/F50A (right).

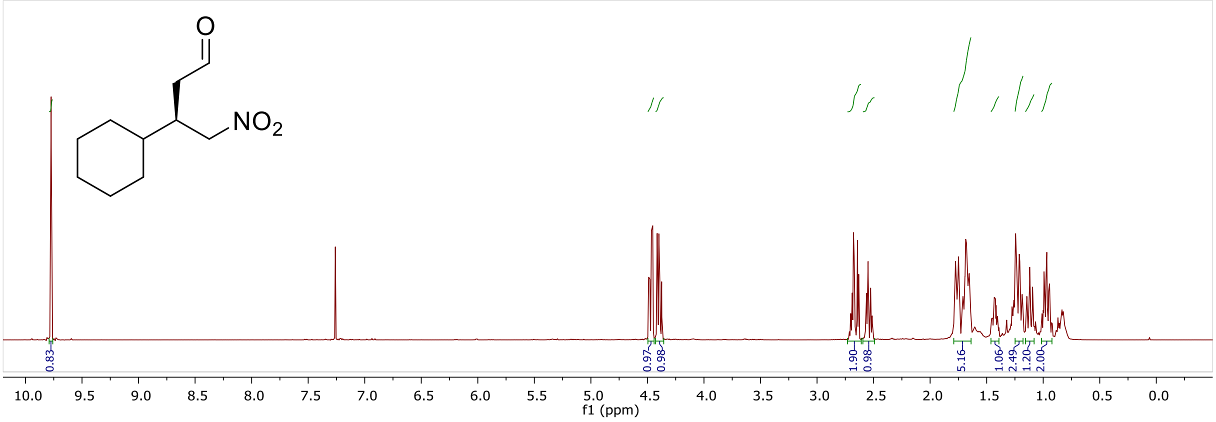

Supplementary Figure 28: ^{1}H NMR spectrum of (*R*)-3-cyclohexyl-4-nitrobutanal (**3f**) synthesized with 4-OT L8Y/M45Y/F50A.

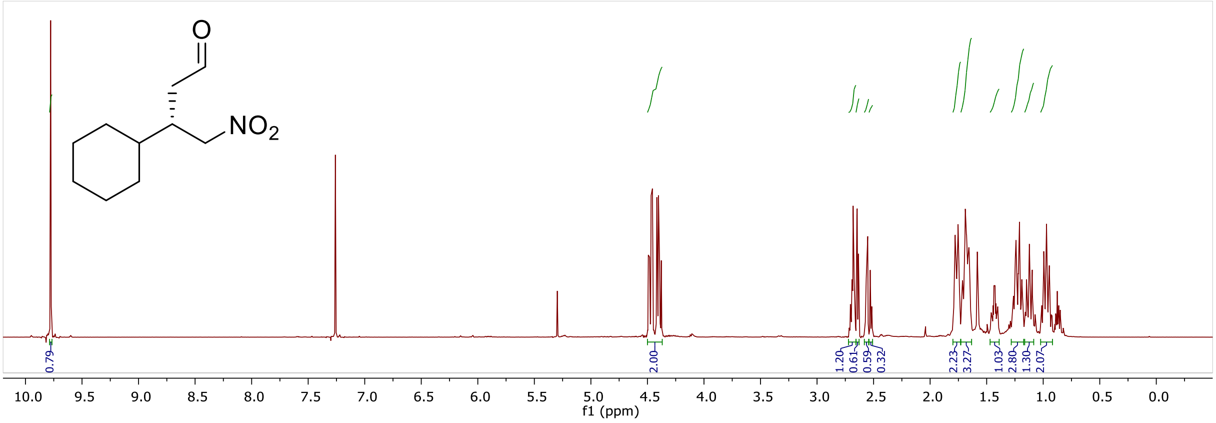

Supplementary Figure 29: ^{1}H NMR spectrum of (*S*)-3-cyclohexyl-4-nitrobutanal (**3f**) synthesized with 4-OT A33D.

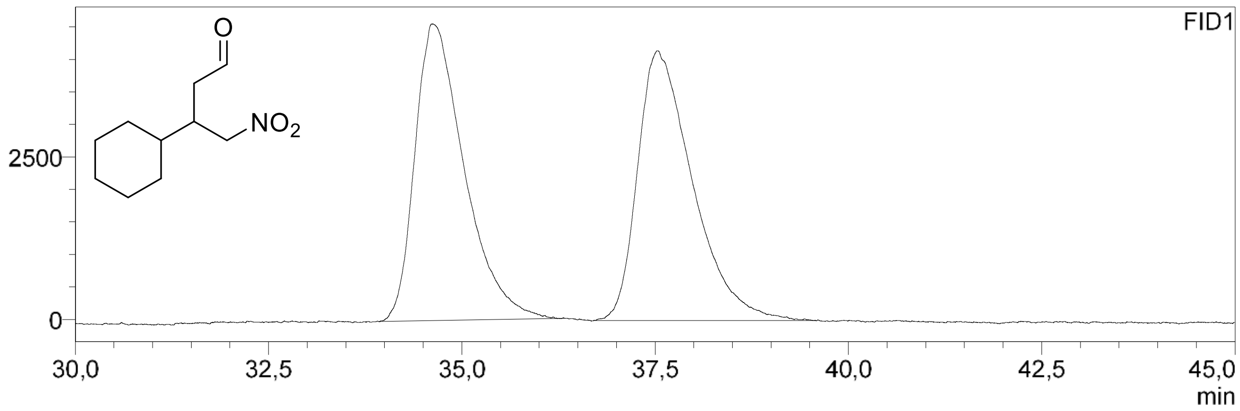

Supplementary Figure 30: GC chromatogram of racemic **3f**.

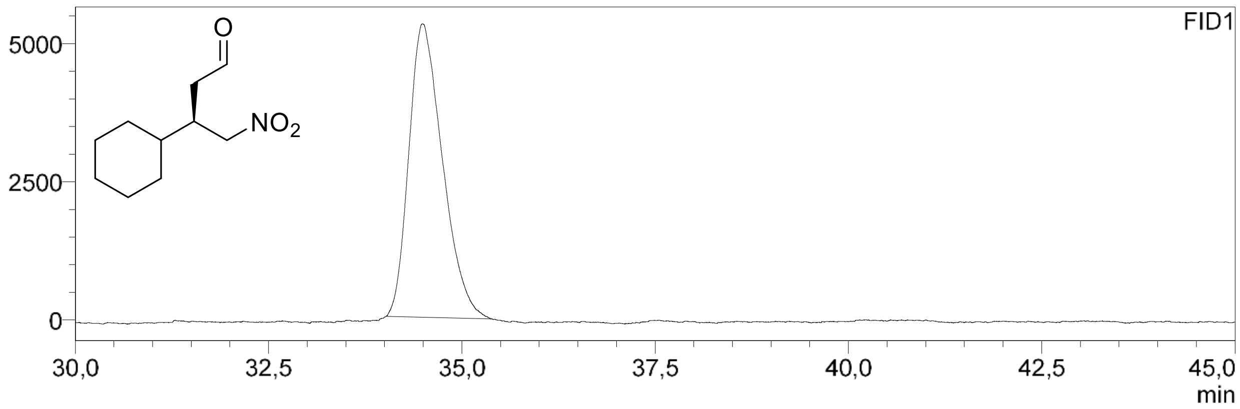

Supplementary Figure 31: GC chromatogram of (*R*)-**3f** obtained with 4-OT L8Y/M45Y/F50A.

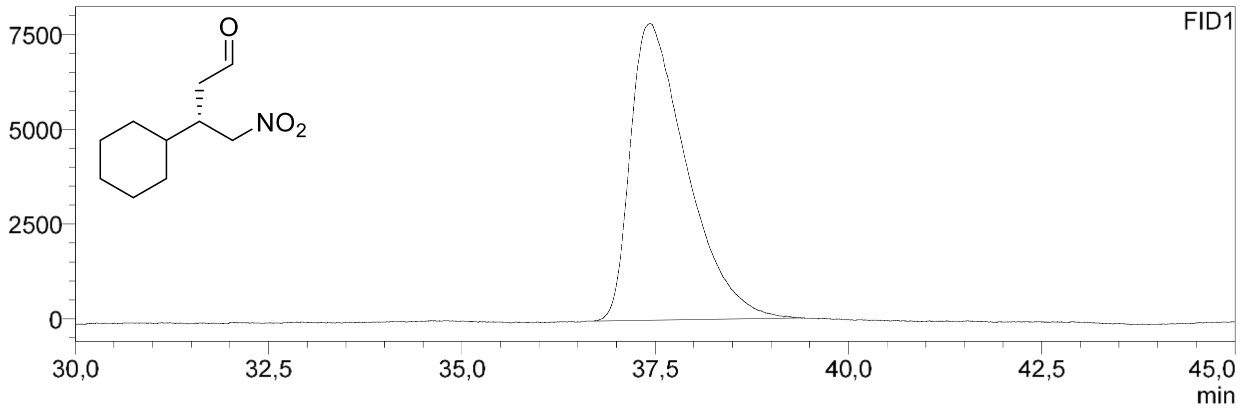

Supplementary Figure 32: GC chromatogram of (*S*)-**3f** obtained with 4-OT A33D.

Supplementary references

[1] S. Maity, T. Naveen, U. Sharma, D. Maiti, *Org. Lett.* **2013**, *15*, 3384–3387.

[2] V. Streitferdt, M. H. Haindl, J. Hioe, F. Morana, P. Renzi, F. von Rekowski, A. Zimmermann, M. Nardi, K. Zeitler, R. M. Gschwind, *Eur. J. Org. Chem.* **2019**, *2019*, 328–337.

[3] N. Wu, B. Wahl, S. Woodward, W. Lewis, *Chem. Eur. J.* **2014**, *20*, 7718–7724.

[4] M. Shin, M. Gu, S. S. Lim, M.-J. Kim, J. Lee, H. Jin, Y. H. Jang, B. Jung, *Eur. J. Org. Chem.* **2018**, *2018*, 3122–3130.

[5] S. Maity, S. Manna, S. Rana, T. Naveen, A. Mallick, D. Maiti, *J. Am. Chem. Soc.* **2013**, *135*, 3355–3358.

[6] N. Duhamel, F. Piano, S. J. Davidson, R. Larcher, B. Fedrizzi, D. Barker, *Tetrahedron Lett.* **2015**, *56*, 1728–1731.

[7] V. Shekhar, D. K. Reddy, Y. Venkateswarlu, *Helv. Chim. Acta* **2012**, *95*, 1593–1599.

[8] M. Riccaboni, E. La Porta, A. Martorana, R. Attanasio, *Tetrahedron* **2010**, *66*, 4032–4039.

[9] K. Ando, K. Yamada, *Green Chem.* **2011**, *13*, 1143.

[10] J. V. Santiago, A. C. B. Burtoloso, *ACS Omega* **2019**, *4*, 159–168.

[11] F. Felluga, G. Pitacco, E. Valentin, C. D. Venneri, *Tetrahedron Asymmetry* **2008**, *19*, 945–955.

[12] W.-X. Liu, S.-K. Chen, J.-M. Tian, Y.-Q. Tu, S.-H. Wang, F.-M. Zhang, *Adv. Synth. Catal.* **2015**, *357*, 3831–3835.

[13] Y. Qiao, J. He, B. Ni, A. D. Headley, *Adv. Synth. Catal.* **2012**, *354*, 2849–2853.

[14] Y. Wang, P. Li, X. Liang, T. Y. Zhang, J. Ye, *Chem. Commun.* **2008**, *10*, 1232.

[15] P. García-García, A. Ladépêche, R. Halder, B. List, *Angew. Chem. Int. Ed.* **2008**, *47*, 4719–4721.

[16] E. Zandvoort, E. M. Geertsema, B.-J. Baas, W. J. Quax, G. J. Poelarends, *Angew. Chem. Int. Ed.* **2012**, *51*, 1240–1243.

[17] L. Biewenga, T. Saravanan, A. Kunzendorf, J.-Y. van der Meer, T. Pijning, P. G. Tepper, R. van Merkerk, S. J. Charnock, A.-M. W. H. Thunnissen, G. J. Poelarends, *ACS Catal.* **2019**, *9*, 1503–1513.

[18] E. Zandvoort, B.-J. Baas, W. J. Quax, G. J. Poelarends, *ChemBioChem* **2011**, *12*, 602–609.

[19] W. J. Waddell, *J. Lab. Clin. Med.* **1956**, *48*, 311–314.

[20] H. Gotoh, H. Ishikawa, Y. Hayashi, *Org. Lett.* **2007**, *9*, 5307–5309.
